# Mechanoluminescence-Enhanced Ammonia Synthesis via Mechanochemical Nitrate Reduction

**DOI:** 10.1021/acsnano.6c05545

**Published:** 2026-06-10

**Authors:** Chunfeng Wang, Gaofeng Lai, Xingyue Yang, Deyu Ruan, Deliang Zhu, Ying Guo, Chunyi Zhi

**Affiliations:** † Guangdong Research Center for Interfacial Engineering of Functional Materials, Guangdong Provincial Key Laboratory of New Energy Materials Service Safety, College of Materials Science and Engineering, 47890 Shenzhen University, Shenzhen 518060, PR China; ‡ Wu Jieh Yee School of Interdisciplinary Studies, 34743 Lingnan University, 8 Castle Peak Road, Tuen Mun, Hong Kong, SAR 999077, China; § Department of Mechanical Engineering, 25809 The University of Hong Kong, Pokfulam, Hong Kong SAR 999077, China

**Keywords:** mechanocatalysis, ammonia synthesis, nitrate reduction, mechanoluminescence, piezophotonic effect

## Abstract

The Haber–Bosch process remains the dominant method for ammonia (NH_3_) synthesis, but it is highly energy-intensive and environmentally burdensome. Nitrate reduction to ammonia (NRA) presents a promising dual-function alternative, enabling both sustainable NH_3_ production and environmental remediation. However, current NRA methods, primarily photocatalytic and electrocatalytic, depend heavily on external energy inputs (light or electricity), limiting their deployment in off-grid or distributed settings. Here, we report a mechanoluminescence (ML)-enhanced mechanocatalysis strategy for mechanically driven NRA using Mn-doped CaZnOS (Mn–CaZnOS) as an efficient mechano-catalyst. Under mechanical stimulation, Mn–CaZnOS generates a synergistic cascade of piezoelectric and photoexcitation effects that facilitate the NRA process. This ML-enhanced system achieves a notable NH_3_ yield rate of 5.4 μmol g^–1^ h^–1^ with exceptional stability over 100 h. Mechanistic investigations, including isotope labeling, kinetic isotope effect, and electron spin resonance, confirm the reaction pathway and identify hydrogenation as the rate-limiting step. Kelvin probe force microscopy and density functional theory calculations reveal that mechanical stimuli induce piezopotential, enriching local NO_3_^–^ concentration and enhancing proton-coupled charge transfer. Additionally, the electronic structure of the active sites is modulated to enhance intermediate adsorption and reduce the energy barrier for NH_3_ formation. This work establishes ML-assisted mechanocatalysis as a mechanically driven platform for sustainable ammonia synthesis and environmental cleanup.

## Graphical abstract

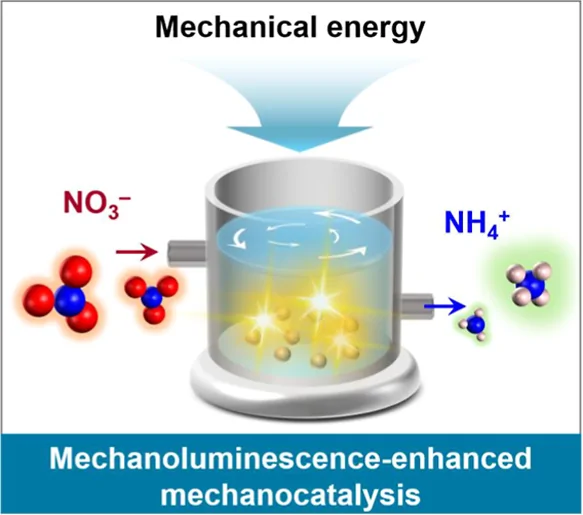

## 1 Introduction

Ammonia (NH_3_) is a cornerstone chemical in global agriculture and industry, widely used in nitrogen-based fertilizers, polymers, and chemical synthesis. It is also gaining attention as a high-density hydrogen carrier with the potential to support carbon-neutral energy systems.^1–5^ Currently, nearly all NH_3_ is produced via the industrial Haber–Bosch (H–B) process, which is energy-intensive and environmentally taxing.^6–10^ Despite the exothermic nature of the reaction (N_2_ + 3H_2_ → 2NH_3_, Δ*H* = −92.44 kJ/mol), this process requires high temperatures and pressures to break the strong NC≡N triple bond and accounts for roughly 1–2% of global energy consumption, contributing to substantial CO_2_ emissions.^11–16^ As a milder alternative, (photo)­electrochemical N_2_ reduction (NRR) has attracted interest for its ability to synthesize NH_3_ directly from N_2_ and H_2_O under ambient conditions.^17–21^ However, the low solubility of N_2_ and its high bond dissociation energy (941 kJ/mol) severely limit the NH_3_ selectivity and yield of NRR, making it less viable than the H–B process.^22–26^ In contrast, nitrate reduction to ammonia (NRA) offers a more sustainable and multifunctional pathway. It not only enables green NH_3_ production but also addresses nitrate pollution, a widespread issue stemming from agricultural runoff, industrial discharge, and urban wastewater.^27–30^ Due to its high solubility, NO_3_^–^ accumulates in aquatic ecosystems, disrupting ecological balance and posing health risks such as methemoglobinemia.^31–34^ NRA thus provides a dual benefit: environmental remediation and decentralized NH_3_ synthesis. However, current NRA technologies, primarily photocatalytic and electrocatalytic, face heavy reliance on external energy inputs (light or electricity), which restrict their scalability and deployment in off-grid or distributed settings (Figure 1a,b).

**Figure 1:**
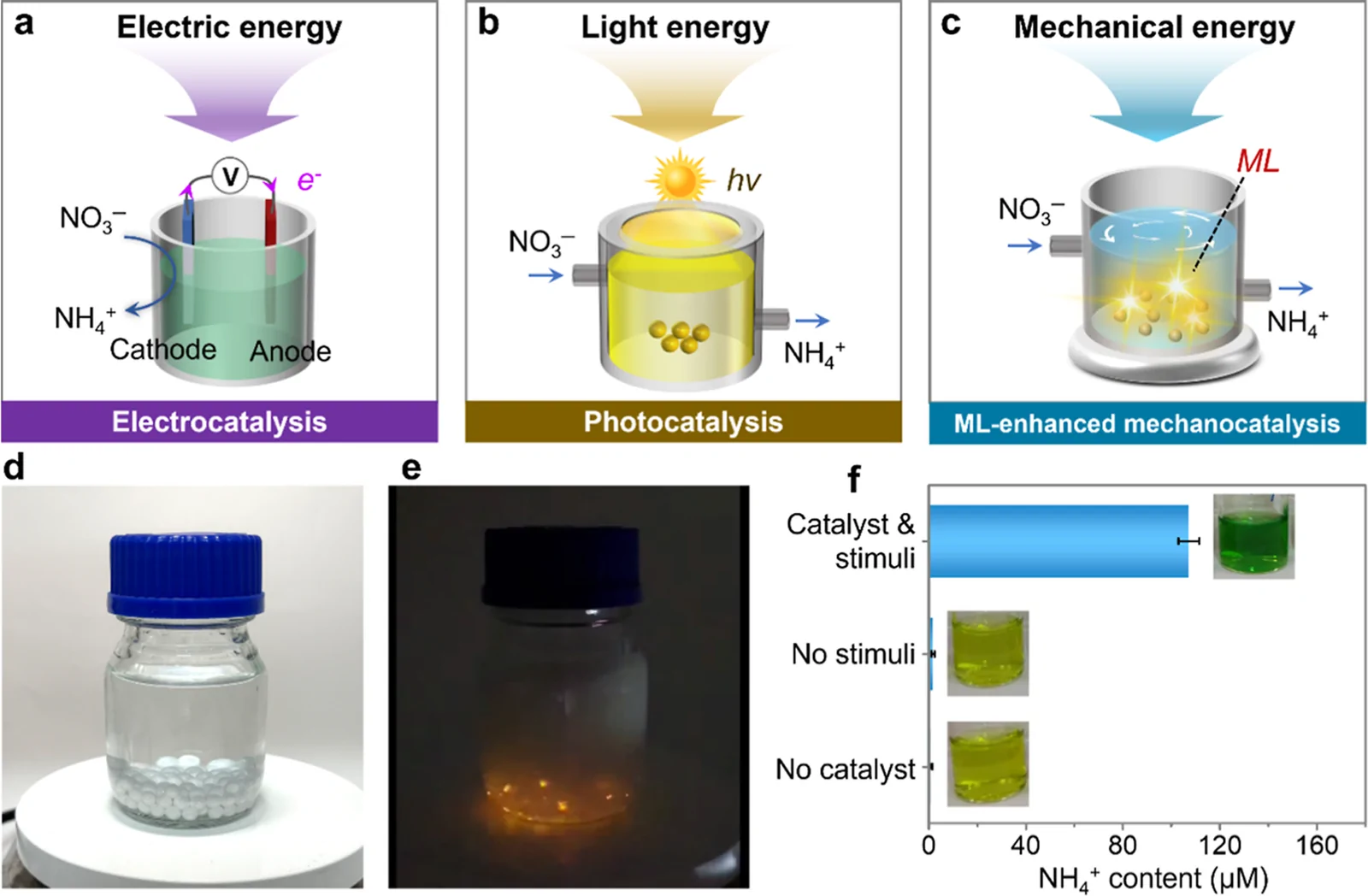
Comparison of different NRA technologies. (a–c) Schematic illustrations of electrocatalytic, photocatalytic, and ML-enhanced mechanocatalytic NRA processes. (d,e) Photos of the static and dynamic NRA devices containing the Mn–CaZnOS catalyst and (f) the resultant NH_3_ content achieved by the ML-enhanced mechanocatalytic NRA process under various conditions.

Mechanically driven catalysis (MDC) has recently emerged as a promising mechanically driven strategy that harnesses pyroelectric, piezoelectric, and ferroelectric materials to convert mechanical, thermal, or light energy into chemical reactions.^35–40^ Among these, piezocatalysis has shown particular promise in environmental remediation and renewable energy conversion.^38^ Piezocatalysts are sensitive to mechanical stimuli such as strain and stress, which induce spatial separation of charge carriers, suppress recombination, and generate a piezo-potential.^41,42^ This process creates strong internal electric fields that continuously drive electrons and holes toward opposite surfaces, a phenomenon widely recognized as the piezoelectric effect.^43–45^ As a result, piezoelectric materials can act as effective catalysts, enabling piezo-catalysis or mechano-catalysis. Compared to photocatalysis, instead of relying on photoinduced charges, mechanocatalysis utilizes mechanically induced charges to drive chemical transformations.^46^ The ability to operate without external energy inputs makes MDC an attractive approach for sustainable catalysis, particularly in distributed or resource-limited environments.

Building on this concept, we propose a mechanochemical strategy for efficient NH_3_ synthesis through mechanoluminescence (ML)-enhanced mechanocatalytic NRA process with Mn-doped CaZnOS (Mn–CaZnOS) catalyst. CaZnOS, a zinc sulfide–based ML material, is highly sensitive to mechanical stimuli and can generate ML behavior without auxiliary energy input.^47,48^ Incorporation of Mn dopants plays a dual role: stabilizing local defects and creating additional defect levels that strengthen ML intensity.^49,50^ This piezophotonic effect arises from the coupling of piezoelectricity and photoexcitation properties. As shown in Figure S1, under mechanical stress, an internal piezopotential tilts the conduction and valence bands of Mn–CaZnOS, reducing the barrier for electron release from trap states into the conduction band. The released electrons recombine nonradiatively with holes, transferring energy to Mn activators and exciting electrons from the ground state ^6^A_1_(^6^S) to the excited ^4^T_1_(^4^G) level.^51^ Relaxation back to the ground state produces orange-red photon emission,^52^ as shown in Figure 1d–e. Collectively, the piezoelectric polarization charges and emitted photons synergistically catalyze the nitrate-to-ammonia conversion. Using this mechanically driven mechanocatalysis strategy, we successfully demonstrated ML-enhanced NH_3_ synthesis with a yield rate of 5.4 μmol g^–1^ h^–1^ and excellent stability (Figure 1f).

## 2 Results and Discussion

### 2.1 Synthesis and Characterization of Mn–CaZnOS

Considering the excellent structural stability and ML performance of zinc sulfide–based compounds, we selected CaZnOS as the host matrix owing to its distinctive lattice topology, consisting of [ZnOS_3_] tetrahedra and [CaO_3_S_3_] octahedra (Figure 2a).^50,53^ This structural framework not only provides a robust basis for efficient ML generation but also allows facile incorporation of luminescent activators such as Mn, Cr, Bi, and Nd. To this end, Mn-doped CaZnOS derivatives were synthesized via a conventional high-temperature solid–state reaction, with Mn introduced at varying doping concentrations. The detailed synthesis procedures for both pristine CaZnOS and Mn–CaZnOS are described in the Experimental Section. Scanning electron microscopy (SEM) images reveal that Mn–CaZnOS particles exhibit irregular morphologies composed of small, roughened spherical grains (Figure S2a,b). Energy-dispersive X-ray spectroscopy (EDS) further confirms the presence of Ca, Zn, O, and S, along with a weak Mn signal, consistent with its relatively low doping level (Figure S2c). As the ML intensity of Mn–CaZnOS is strongly dependent on Mn content, which plays a critical role in activating the ML effect, a series of samples with different Mn weight ratios was prepared to identify the optimal doping concentration. X-ray photoelectron spectroscopy (XPS) was employed to investigate the chemical states and electronic configurations of the as-prepared samples. As shown in Figures 2b, S3a, in comparison with pristine CaZnOS, Mn–CaZnOS exhibits a distinct Mn signal in addition to the characteristic peaks of Ca, Zn, O, and S, confirming the successful incorporation of Mn dopants into the CaZnOS matrix. These results are consistent with the EDS analysis. The Mn contents in the Mn–CaZnOS samples were further quantified by inductively coupled plasma mass spectrometry (ICP–MS), as summarized in Table S1. To gain deeper insight into the crystal structure and possible Mn-substitution sites, Rietveld refinement was performed using the GSAS program. The refinement results, shown in Figure 2c, yielded reasonable R-factors, confirming the reliability of the structural model. The refined structure demonstrates that Mn–CaZnOS crystallizes in the noncentrosymmetric hexagonal space group *P*6_3_ *mc* (no. 186), composed of alternating [ZnOS_3_] tetrahedra and [CaO_3_S_3_] octahedra.^48^ The lack of inversion symmetry in these distorted polyhedra promotes piezoelectric polarization under applied stress, thereby establishing a strong structural foundation for efficient ML. In addition, the alternately stacked tetrahedral and octahedral layers along the *c*-axis form a layered structure that facilitates electronic transport. Considering the ionic radii and coordination environments of Ca^2+^ (*r* = 1.00 Å, CN = 6), Zn^2+^ (*r* = 0.60 Å, CN = 6), Mn^2+^ (*r* = 0.66 Å, CN = 4; *r* = 0.83 Å, CN = 6), and Mn^4+^ (*r* = 0.53 Å, CN = 6), and applying the Hume–Rothery rules (Supporting Information), which stipulate that the acceptable ionic radius mismatch should not exceed 30%, the calculated mismatch values Δ*r*(Mn^2+^–Ca^2+^) = 17% and Δ*r*(Mn^2+^–Zn^2+^) = 10% suggest that Mn^2+^ can feasibly substitute for both Ca^2+^ and Zn^2+^ sites.

**Figure 2:**
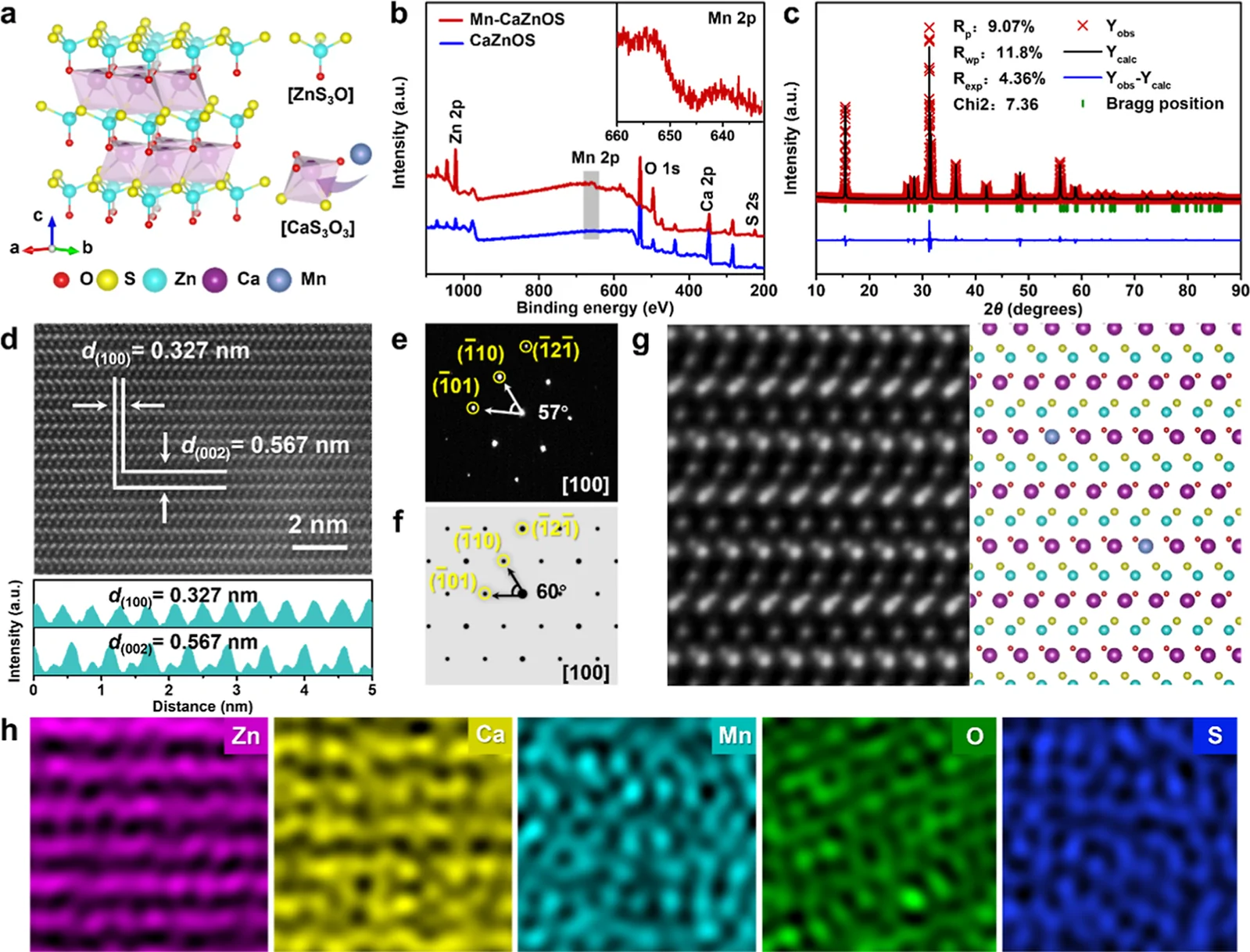
Structural and morphological characterization. (a) Crystal structure of Mn–CaZnOS and the Mn doping site, (b) Survey XPS spectra of CaZnOS and Mn–CaZnOS, (c) XRD pattern and the Rietveld refinement of Mn–CaZnOS. (d) HAADF-STEM image and the intensity profiles of the (100) and (002) planes in Mn–CaZnOS. (e,f) the FFT pattern and the corresponding simulated diffraction pattern of Mn–CaZnOS. (g) The atomic-resolution HAADF-STEM image and the corresponding crystal structure model of Mn–CaZnOS. (h) The EDX elemental mapping images of Mn–CaZnOS.

Further structural evidence was obtained from high-angle annular dark-field scanning transmission electron microscopy (HAADF-STEM). A representative image of an individual Mn–CaZnOS particle (Figure 2d) shows a highly ordered atomic arrangement along the [100] zone axis. Two distinct lattice fringes with interplanar spacings of 0.327 and 0.567 nm correspond to the (100) and (200) planes, respectively. These values slightly deviate from the standard references of 0.325 and 0.570 nm (PDF#01-076-3819), as highlighted by the relative intensity profiles, indicating lattice distortion induced by Mn doping. The fast Fourier transform (FFT) pattern (Figure 2e) further confirms the single-crystalline nature of the as-prepared Mn–CaZnOS, displaying diffraction spots consistent with the simulated pattern (Figure 2f). The diffraction streaks corresponding to the (1̅10), (11̅0), and (1̅21̅) planes verify the high degree of atomic ordering in Mn–CaZnOS. Interestingly, the angle between the (1̅10) and (1̅21̅) planes is approximately 57°, slightly smaller than the theoretical value of 60°, providing additional evidence of Mn-induced lattice distortion. This observation is consistent with the results shown in Figure 2d. Despite the slight lattice distortion, the overall atomic arrangement of Mn–CaZnOS remains in good agreement with the standard hexagonal model (Figure 2g). Atomic-resolution EDS elemental mapping further demonstrates the homogeneous distribution of Ca, Zn, Mn, S, and O throughout the lattice, with Zn atoms clearly occupying their expected crystallographic sites. These findings collectively confirm the successful synthesis of Mn-doped CaZnOS with well-defined structural features, uniform elemental distribution, and subtle lattice distortions induced by Mn incorporation, all of which provide a robust foundation for subsequent ML performance.

### 2.2 ML Performance of Mn-Doped CaZnOS

The ML performance of Mn–CaZnOS was systematically evaluated by sealing the powders between two square polyethylene terephthalate (PET) sheets (3.5 × 3.5 cm^2^). As illustrated in Figure 3a, writing on the Mn–CaZnOS sheet with a hard pen tip produced vivid orange-red luminescence that was readily captured by a digital camera. Mapping the spatial distribution of these traces enabled the reconstruction of a three-dimensional contour of stress-induced ML intensity (Figure 3b), clearly demonstrating that even subtle variations in writing pressure were faithfully reflected in the emission intensity. In addition, Mn–CaZnOS powders displayed bright luminescence when rubbed with a glass rod, while vigorous shaking of a glass vial containing ZrO_2_ balls coated with Mn–CaZnOS powder generated striking sparks, as shown in Movies S1 and S2. These qualitative demonstrations highlight the strong stress-responsive luminescence of Mn–CaZnOS and its potential for ML-enhanced NRA process. Furthermore, the ML behavior of Mn–CaZnOS was quantitatively characterized with a custom-built setup integrating a linear motor, dynamometer, and fiber-optic spectrometer and the light emission spectra were recorded under controlled loading conditions. As shown in Figure 3c, the ML intensity increased with Mn content and reached a maximum at 3.0 at % under a mechanical load of 20 N. Beyond this level, excessive doping likely induced concentration quenching, resulting in reduced emission intensity. All samples exhibited a broad visible emission band spanning 550–750 nm with a dominant peak at 600 nm, which remained nearly unchanged across different Mn levels, indicating that dopant concentration primarily modulated emission intensity rather than spectral position. The optimized composition (3.0 wt % Mn) was further examined for force-dependent ML response. As shown in Figure 3d, detectable luminescence was observed at forces as low as 1 N, and the emission intensity increased linearly with applied load from 1 to 30 N, yielding an excellent correlation coefficient of 0.997. This linearity underscores the high force sensitivity and reproducibility of Mn–CaZnOS. The corresponding emission spectrum was converted into CIE chromaticity coordinates (0.643, 0.354), confirming a stable orange-red emission (Figure 3e). Furthermore, the ML intensity remained nearly unchanged after 600 loading–unloading cycles (Figure 3f), demonstrating the outstanding mechanical durability and operational stability of Mn–CaZnOS, both of which are critical for its potential applications in mechanochemical energy conversion.

**Figure 3:**
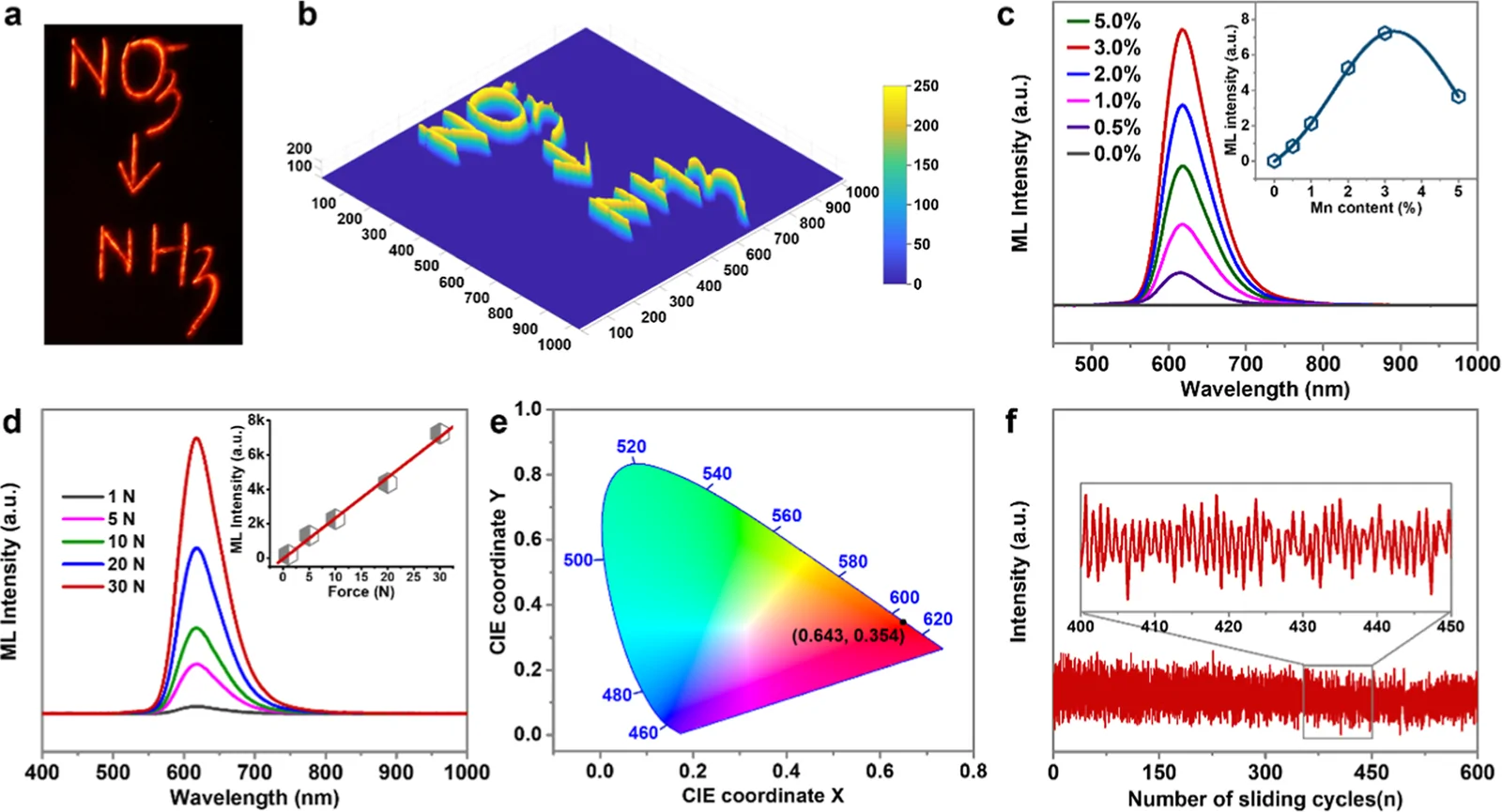
ML performance. (a,b) ML photographs of Mn–CaZnOS and 3D visualization of relative force distribution extracted from (a) based on the gray scale value. (c) The ML intensity diagram of Mn–CaZnOS with different Mn doping contents under the applied force of 30 N. (d) The ML spectra of Mn–CaZnOS subjected to various dynamic loads (Insert: Quantitative relationship between the ML intensity and applied load). (e) CIE chromaticity diagram and cycle stability test for Mn–CaZnOS. (f) ML stability and repeatability over 600 loading cycles at constant pressure.

### 2.3 ML-Enhanced NRA Performance over Mn-Doped CaZnOS Catalyst

The nitrate reduction reaction (NRA) performance of the Mn–CaZnOS catalyst was investigated under ambient conditions using a flask containing ZrO_2_ balls and the optimal Mn–CaZnOS catalyst with 3 wt % Mn content in an aqueous NO_3_^–^ electrolyte (Figure S4 and movie S3). The reaction products, including NO_2_^–^, NH_3_, and N_2_H_4_, were quantified by ultraviolet–visible (UV–Vis) spectroscopy using standard calibration curves (Figures S5–S8). Since CaZnOS is a p-type semiconductor with potential photocatalytic activity toward NRA, we examined the photocatalytic performance of Mn–CaZnOS under simulated sunlight in the absence of mechanical stirring, using 0.1 M NO_3_^–^ in deionized (DI) water as the electrolyte. As shown in Figures 4a and S9, the NH_3_ concentration gradually increased from 25.2 to 32.2 μM with increasing light intensity, confirming the photocatalytic activity of Mn–CaZnOS toward NRA. Obvisously, when the catalyst was subjected to intense mechanical stimuli in the dark, the NH_3_ concentration rose sharply to 100.1 μM, significantly higher than under illumination. This result provides compelling evidence that the ML effect of Mn–CaZnOS plays the dominant role in catalyzing nitrate-to-ammonia conversion. To further validate this conclusion, a series of control experiments was conducted in the dark (Figures 4b and S10). In the absence of either Mn–CaZnOS or mechanical agitation, the NH_3_ yield was negligible compared with that obtained under external mechanical stimuli. Given that NRA is a proton-coupled reaction, highlighting the critical role of H^+^, we next employed phosphate-buffered saline (PBS, pH = 7.2) as the electrolyte, which provides a more stable proton concentration than DI water. As expected, the resultant NH_3_ in PBS was higher than in DI water, underscoring the importance of proton availability in facilitating NRA. It should be noted that strongly acidic electrolytes were not considered, as Mn–CaZnOS, being an oxide-based material, is unstable under acidic conditions.

**Figure 4:**
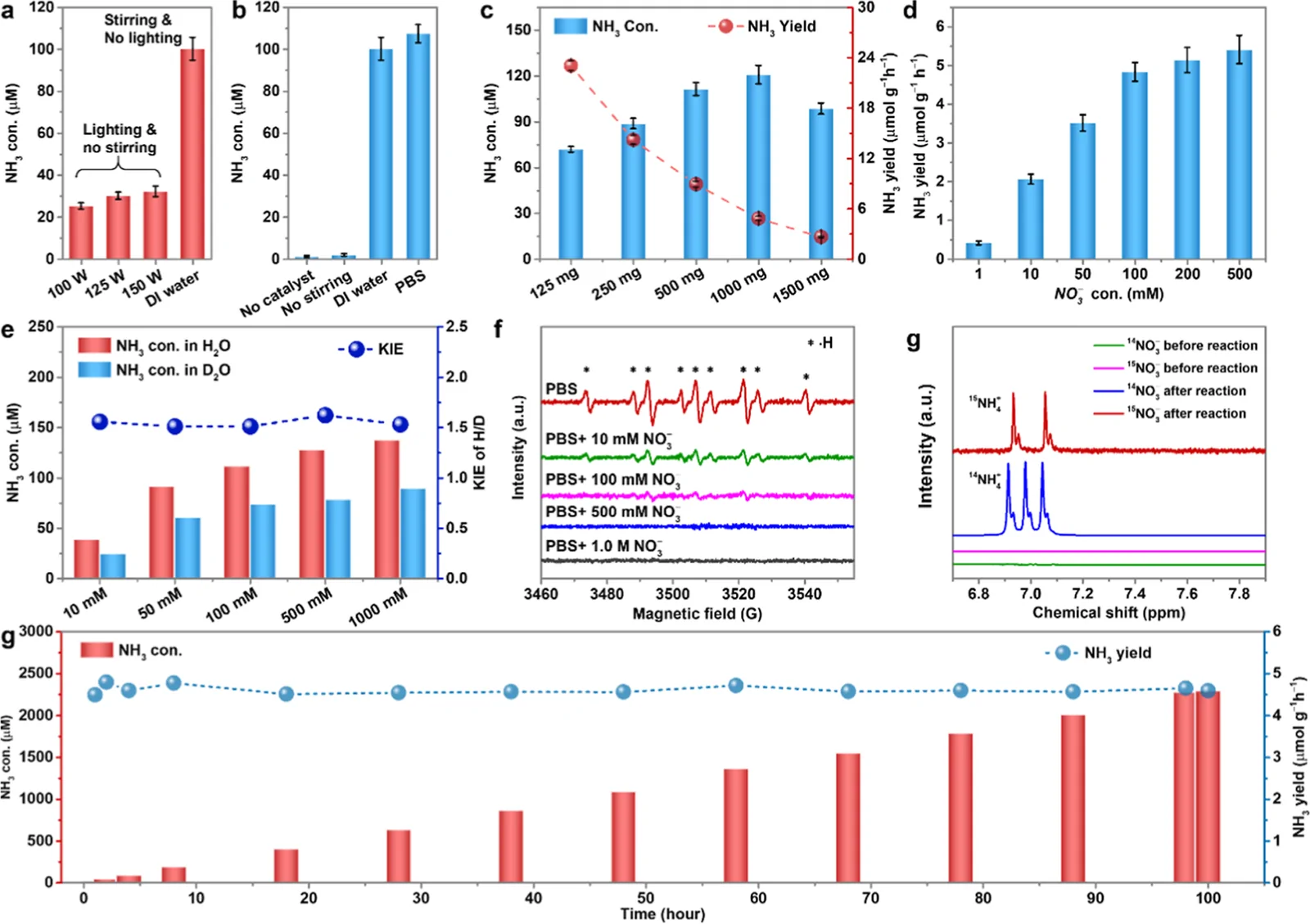
ML-enhanced NRA performance over the Mn–CaZnOS catalyst. (a) NH_3_ concentration achieved at ultrasonic treatment with different powers using DI water as the electrolyte. (b) NH_3_ concentration without catalyst or stirring treatment or using DI water or PBS electrolytes. (c) NH_3_ production of Mn–CaZnOS with different weights in PBS electrolyte. (d) NH_3_ yields of Mn–CaZnOS with different NO_3_^–^ concentrations. (e) NH_3_ concentration and KIE investigation of H/D over Mn–CaZnOS with various NO_3_^–^ concentrations. (f) ESR spectra of NRA catalyzed by Mn–CaZnOS with or without NO_3_^–^ using DMPO as the radical trapping reagent. (g) ^1^H NMR analysis of the resultant NH_4_^+^ with/without the ^15^NO_3_^–^ and ^14^NO_3_^–^ electrolytes. (h) Long-term NRA test of Mn–CaZnOS over 100 h.

Having confirmed the suitability of PBS as a favorable electrolyte for the NH_3_ production, we further investigated the influence of catalyst loading on NRA performance. As shown in Figure 4c, the NH_3_ concentration increased with increasing Mn–CaZnOS mass, reaching a maximum of 120.7 μM at 1000 mg of catalyst. However, the normalized NH_3_ yield rate decreased from 23.0 to 2.6 μ mol g^–1^ h^–1^ as the catalyst mass increased, indicating that while higher catalyst loading enhances overall NH_3_ production, it does not proportionally improve the yield rate per unit mass. This decrease in mass-normalized yield stems from the dilution of mechanical energy input per unit mass. Furthermore, elevated packing density exacerbates particle agglomeration and fluid dampening, causing nonuniform stress distribution and restricting reactant mass transfer, which ultimately limits piezo-potential generation and active site utilization efficiency. Notably, as shown in Figure S11, pristine CaZnOS exhibited a significantly lower NH_3_ content compared with Mn-doped CaZnOS, owing to the absence of ML property in CaZnOS (Figure 3c), thereby highlighting the critical role of the in situ generated ML effect of Mn–CaZnOS in improving mechanocatalytic NH_3_ production. Potential byproducts such as N_2_, NO_2_^–^ and N_2_H_4_ were also quantified by gas chromatograph and UV–Vis spectroscopy (Figures S12–S14). Their concentrations were negligible, demonstrating the excellent selectivity of Mn–CaZnOS for target NO_3_^–^-to-NH_3_ conversion. Based on these results, 1000 mg of Mn–CaZnOS was selected for subsequent experiments. We then examined the effect of varying NO_3_^–^ concentrations on NRA performance. As shown in Figures 4d and S15, when the NO_3_^–^ concentration was increased from 1 mM to 500 mM, the absorbance intensity of the resultant electrolytes rose accordingly, with the significant improvement at lower NO_3_^–^ concentrations. The NH_3_ yield rate also increased, reaching a maximum of 5.4 μmol g^–1^ h^–1^. However, no linear correlation was observed between NH_3_ yield rate and catalyst mass, further emphasizing the complex interplay between catalyst loading, reactant concentration, and reaction kinetics.

As is well-known, the NRA process follows the proton-coupled charge transfer pathway (NO_3_^–^ + 9H^+^ + 8e^–^ → NH_3_ + H_2_O), highlighting the critical roles of adsorbed hydrogen (H_ads_) and NO_3_^–^ in the reaction mechanism. To probe the protonation process, we examined the kinetic isotope effect (KIE), defined as the ratio of NH_3_ yield rates in H_2_O and D_2_O (Figures 4e and S16). The average KIE value of 1.55 indicates that hydrogenation is the rate-determining step (RDS) in the NRA process. This suggests that Mn–CaZnOS facilitates H_2_O dissociation, thereby enhancing protonation of intermediates and accelerating reaction kinetics. To directly confirm the presence of H_ads_, electron spin resonance (ESR) spectroscopy was performed with different NO_3_^–^ concentrations using 5,5-dimethyl-1-pyrroline-*N*-oxide (DMPO) as the radical trapping agent. As shown in Figure 4f, nine strong peaks with an intensity ratio of 1:2 were observed in the ESR spectrum of Mn–CaZnOS in pure PBS, corresponding to the DMPO-H spin adduct, providing direct evidence of H_ads_ formation. The DMPO-H signal intensity decreased sharply with increasing NO_3_^–^ concentration and disappeared completely at 1.0 M NO_3_^–^, indicating that H_ads_ generated by water splitting were consumed during NRA. This demonstrates the critical role of H_ads_ equilibrium in sustaining the reaction. In addition to confirming the role of adsorbed hydrogen, isotope-labeling experiments were conducted to trace the nitrogen source of the NH_3_ product. Figure 4g shows the ^1^H NMR spectra of NH_4_^+^ obtained from NRA using ^15^NO_3_^–^ and ^14^NO_3_^–^ electrolytes. Triplet peaks corresponding to ^14^NH_4_^+^ and doublet peaks corresponding to ^15^NH_4_^+^ were detected when ^15^NO_3_^–^ and ^14^NO_3_^–^ were used, respectively, while no peaks were observed in the absence of labeled NO_3_^–^. These results confirm that all NH_3_ products indeed originated from the NRA process catalyzed by Mn–CaZnOS. Finally, the long-term stability of Mn–CaZnOS was evaluated by continuous NRA operation for 100 h. As shown in Figures 4g and S17, the NH_3_ concentration increased linearly with time, while the NH_3_ yield rate remained stable at approximately 4.61 μmol g^–1^ h^–1^ without significant fluctuation. This demonstrates the excellent durability and operational stability of Mn–CaZnOS, further supporting its potential as a robust piezocatalyst for ML-enhanced NH_3_ synthesis. The XRD pattern, SEM and HAADF-STEM images in Figures S18 and 19 exhibits a well preserved crystalline and microstructures of the Mn–CaZnOS mechano-catalyst after the long-term test, consistent with those before the NRA test. Besides, the FFT pattern confirms the single-crystalline feature of Mn–CaZnOS after the NRA test (Figure S19d). All these collectively evidence the decent structural stability of the Mn–CaZnOS catalyst. ML-enhanced NH_3_ production via mechanochemical nitrate reduction remains scarcely explored (Table S2). Our work establishes a distinct design principle for mechanically driven catalysis, using ML to activate reactions without external energy, enabling off-grid ammonia synthesis and motivating further optimization for improved performance and scalability.

### 2.4 Understanding of the ML-Enhanced NRA Mechanisms

To gain deeper insight into the ML-enhanced NRA mechanism over the Mn–CaZnOS piezocatalyst, density functional theory (DFT) calculations were performed on the (100) facets based on previous experimental observations. Considering the possible substitution of Mn for Zn or Ca and the presence of O and S vacancies in the Mn–CaZnOS lattice, seven structural models were constructed (Table S3). Among them, the Mn–CaZnOS–Ca-Vs model exhibited the lowest total energy, indicating its superior thermodynamic stability (Figure S20); this optimized structure was therefore selected for further mechanistic investigation. Since the work function (W) is a critical parameter for predicting charge transfer during catalytic NRA, we evaluated this factor for both the relaxed and mechanically pressed Mn–CaZnOS (see Supporting Information for details). As shown in Figure S21, the pressed Mn–CaZnOS exhibited a lower work function (*W*_press_ = 0.658 eV) compared with the relaxed state (*W*_relax_ = 0.572 eV), reflecting its reduced electrostatic potential. This difference in work function between the two states induces a polarization effect and, thus, in situ generates a piezopotential at the contact points under mechanical stress. Consequently, electrons are driven from the relaxed to the pressed regions, thereby facilitating proton-coupled electron transfer for the NRA process at these interfacial sites.^54^

To experimentally verify this piezoelectric effect, Kelvin probe force microscopy (KPFM) was employed, as it provides spatially resolved measurements of surface potential (contact potential difference, CPD) and work function in semiconductors. As shown in Figure 5a,b, the topographic image and corresponding CPD profiles of Mn–CaZnOS reveal distinct contrasts in surface potential across different domains, further confirmed by the CPD distribution along the white dashed line in Figure 5c. These results, combined with theoretical calculations, conclusively demonstrate that intense mechanical stimuli induce an electric field on the Mn–CaZnOS surface in favor of the NRA process. To further probe the role of this field, molecular dynamics (MD) simulations were performed under different electric field conditions (Figure 5d–e). As shown in Figure 5f, in the absence of an applied field, NO_3_^–^ anions were uniformly distributed in the bulk electrolyte. In contrast, under an applied field, a pronounced accumulation of NO_3_^–^ anions was observed near the Mn–CaZnOS surface. This finding indicates that the in situ generated electric field effectively regulates the charge distribution within the inner Helmholtz plane, enriches the local NO_3_^–^ concentration at the catalyst surface, and thereby benefits the NO_3_^–^-to-NH_3_ transformation.

**Figure 5:**
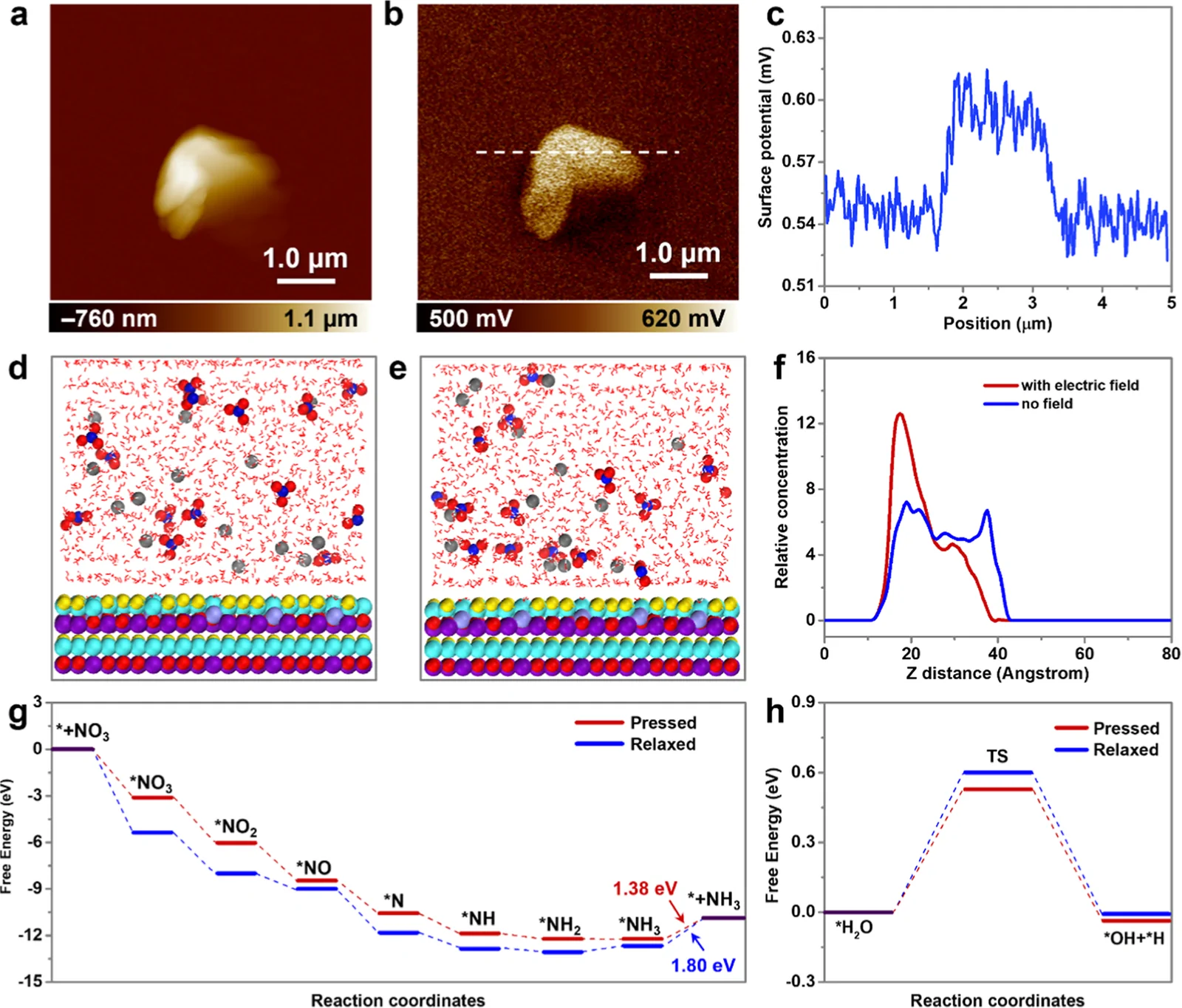
ML-enhanced NRA mechanism. (a) Topography and (b) CPD profiles of the Mn–CaZnOS catalyst. (c) the corresponding CPD distribution along the white disk line in Figure 5b. (d,e) MD simulation of the pressed Mn–CaZnOS in the electrolyte with K^+^, NO_3_^–^, and water molecules without (d) or with (e) the applied electric field of −0.8 V and (f) the relative density profiles of NO_3_^–^ along the *Z* axis direction. (d) The Gibbs free energies of the NRA process and (e) transition states of H_2_O dissociation over the relaxed and pressed Mn–CaZnOS.

To better understand the reaction pathway and elucidate the relationship between intermediate adsorption and the NRA activity, DFT calculations were carried out for both relaxed and pressed Mn–CaZnOS models. Optimized adsorption configurations for each intermediate are summarized in Tables S4 and S5. The NRA proceeds through a sequence of deoxygenation steps (*NO_3_ → *NO_2_ → *NO → N), followed by hydrogenation steps (N → *NH → *NH_2_ → *NH_3_), consistent with previous reports. All possible adsorption sites and configurations were considered, and the most stable structures with the lowest free energies were used to construct the reaction pathway. As shown in Figure 5g, except for the NH_3_ evolution step, the free energy profiles of all intermediates decrease monotonically, indicating that the NRA process over both relaxed and pressed Mn–CaZnOS can proceed spontaneously via electron transfer and hydrogenation. Notably, the pressed Mn–CaZnOS consistently exhibits lower absorption energies for intermediates in favor of the desorption behavior, resulting in a narrower energy span and accelerated reaction kinetics. This enhancement is attributed to the downshifted d-band center of the Mn–CaZnOS due to the external mechanical stimuli (Figure S22), which strengthens the interaction with intermediates. Nevertheless, for both models, the NH_3_ desorption step remains energetically uphill, identifying it as the rate-determining step (RDS). Importantly, the desorption barrier for *NH_3_ on the pressed Mn–CaZnOS surface is only 1.38 eV, significantly lower than that of the relaxed state, suggesting that mechanical stress facilitates NH_3_ release and thus accelerates overall NRA kinetics. In addition to NRA, the competing hydrogen evolution reaction (HER) was also considered. The energy barrier for H_2_O dissociation, a key step in both NRA and HER, was calculated for both models. As shown in Figure 5h, the pressed Mn–CaZnOS exhibits a lower barrier (0.53 eV) compared with the relaxed state, indicating enhanced water splitting and proton generation. This facilitated proton supply further promotes the hydrogenation steps of the NO_3_^–^-to-NH_3_ conversion, thereby reinforcing the mechanistic role of mechanical stimuli in boosting NRA activity.

## 3 Conclusions

In summary, we have demonstrated an ML-enhanced mechanochemical strategy for sustainable ammonia synthesis via nitrate reduction, employing Mn-doped CaZnOS (Mn–CaZnOS) as a mechanically driven mechano-catalyst. This approach effectively addresses the limitations of current NRA routes by eliminating dependence on external energy inputs such as light or electricity. The optimized Mn–CaZnOS catalyst (3 wt % Mn) exhibited robust and stable ML performance, a linear force-dependent luminescence response, and impressive durability over extended operation. Under mechanical stimulation, the catalyst achieved a high NH_3_ yield rate of 5.4 μmol g^–1^ h^–1^ with excellent selectivity and stability exceeding 100 h. Mechanistic investigations, including isotope labeling, ESR, KIE, and DFT calculations, confirmed hydrogenation as the rate-determining step and revealed that mechanical stress significantly lowers the energy barrier for NH_3_ desorption. Additionally, KPFM and MD simulations reveal that in situ piezopotential enriches local NO_3_^–^ concentration and promotes interfacial charge transfer. These findings establish ML-assisted mechanocatalysis as an energy-efficient platform for green NH_3_ synthesis, offering dual benefits of environmental remediation and chemical synthesis. This strategy unlocks new opportunities for designing mechanically driven catalytic systems and provides a foundation for future development of piezo-responsive materials in green chemistry and decentralized nitrogen conversion technologies.

## 4 Experimental Section

### 4.1 Synthesis of the Pristine CaZnOS and Mn-Doped CaZnOS

The Mn-doped CaZnOS (Mn–CaZnOS) phosphors were synthesized via a conventional high-temperature solid–state reaction. Stoichiometric amounts of CaCO_3_, ZnS, and MnCO_3_ were weighed according to the desired Ca/Zn:Mn molar ratio and thoroughly mixed in an agate mortar with ethanol as a dispersing medium until the ethanol nearly evaporated. The homogeneous mixture was transferred into a corundum boat and dried at 80 °C for 4 h, followed by calcination at 1100 °C for 4 h in a horizontal tube furnace under an 80 sccm argon flow. The heating profile involved ramping from room temperature (∼20 °C) to 800 °C at 10 °C·min^–1^, holding at 800 °C for 1 h, increasing to 1100 °C at 5 °C·min^–1^, and maintaining that temperature for 4 h before cooling naturally to room temperature. The resulting product was ground into fine powder using a ball mill. Pristine CaZnOS was synthesized using the same procedure without the Mn precursor.

### 4.2 Characterizations

The crystalline structures, morphologies, and microstructures of the samples were examined by XRD using a Bruker D2 Phaser diffractometer with Cu Kα radiation (λ = 1.54 Å), field-emission scanning electron microscopy (FEI Quanta 450 FEG), and JEOL-2001F field-emission TEM. The structural features and chemical states of the samples were analyzed by Fourier transform infrared spectroscopy (FTIR, Prestige-21). The surface chemical states and compositions of the obtained products were characterized by XPS (ESCALB 250) with an Al Kα X-ray source (E = 1486.6 eV). ^1^H NMR spectra were collected using a Bruker 600 MHz ASCEND AVANCE III HD nuclear magnetic resonance system (NMR-600). UV–Vis spectroscopy was performed with a Cary 5000 UV–Vis–NIR spectrometer (Agilent). Differential electrochemical mass spectrometry (DEMS) was provided by Linglu Instruments (Shanghai) Co. Ltd. The geometric structures of the samples were determined by X-ray absorption near-edge structure (XANES) analysis. The ML performances were evaluated using a custom-built apparatus consisting of a linear motor, dynamometer, and fiber-optic spectrometer (Ocean QE65 Pro).

### 4.3 ML-Enhanced NRA Test

Deionized (DI) water and phosphate-buffered saline (PBS) solution (40 mL) were employed as electrolytes containing various NO_3_^–^ concentrations. ZrO_2_ balls of varying diameters (2 mm, 4 mm, 5 mm, 10 mm, and 15 mm) were used to generate mechanical stimuli. The ML-enhanced NRA experiments were carried out in sealed glass flasks containing the Mn–CaZnOS catalyst with different weights, 40 mL of electrolyte, and ZrO_2_ balls (20 g, equal mass for each diameter size), under magnetic stirring at 800 rpm for 1 h in dark conditions. For durability evaluation, long-term NRA testing was performed using 200 mL of PBS electrolyte under identical stirring conditions (800 rpm for 1 h in the dark).

### 4.4 Computational Details

MD simulations were conducted with COMPASS III force field using the Forcite module to study the system in the presence of NO_3_^–^, K^+^, and H_2_O in a rectangular box with length scales of 39.04 Å × 40.17 Å × 41.06 Å. After geometry optimization, were performed under the *NVT* ensemble (*T* = 298.0 K) with a time step of 1 fs and a total simulation time of 1 ns, during which simulation trajectories were recorded every 5000 steps. All calculations were performed using density functional theory (DFT) within the DMol3 package of the Materials Studio software.

## Supplementary Material



## Notes

Additional experiments for NRA measurements and, the determination of nitrate and nitrite; additional SEM images, TEM images, XRD patterns, XPS spectra and Raman spectra. UV–vis absorption curves and calibration lines for ammonia, byproducts; calculated ammonia yields and FEs; tables for DFT data (PDF)
