# Supplementary material for: Mechanoluminescence-Enhanced Ammonia Synthesis via Mechanochemical Nitrate Reduction
Source: ACS Nano. 2026 Jun 10;20(24):17695–704. doi: 10.1021/acsnano.6c05545 (PMC13557438; doi:10.1021/acsnano.6c05545)
Supplement: Supplementary file 1 [file nn6c05545_si_001.pdf]

Supplementary Information

Mechanoluminescence-Enhanced Ammonia Synthesis via Mechanochemical Nitrate Reduction

Chunfeng Wang[a], Gaofeng Lai[a], Xingyue Yang[b,c], Deyu Ruan[a], Deliang Zhu[a], Ying Guo[b]*, Chunyi Zhi[c]*

[a]Guangdong Research Center for Interfacial Engineering of Functional Materials, Guangdong Provincial Key Laboratory of New Energy Materials Service Safety, College of Materials Science and Engineering, Shenzhen University, Shenzhen 518060, PR China.

[b]Wu Jieh Yee School of Interdisciplinary Studies, Lingnan University, 8 Castle Peak Road, Tuen Mun, Hong Kong, SAR, 999077 China

[c]Department of Mechanical Engineering, The University of Hong Kong, Pokfulam, Hong Kong SAR, 999077 China

Corresponding author: Dr. Ying Guo and Prof. Chunyi Zhi

Email: ying.guo@ln.edu.hk; cyzhi@hku.hk

Experiments and methods

S1. Experiment section

Chemicals

Phosphate buffer solution, potassium sulfate (K_2SO_4, 99.99%, metals basis), deuterium oxide (D_2O, 99.9 atom % D), hydrochloric acid (HCl, 37%), potassium nitrate (KNO_3, 99.99%, metals basis), potassium nitrate-^{15}N ($K^{15}NO_3$, 99 atom % ^{15}N), ammonia sulfate(($^{15}NH_4)_2SO_4$, 99 atom % ^{15}N), p-Dimethylaminobenzaldehyde ($C_9H_{11}NO$), sodium hydroxide (NaOH, > 98%), salicylic acid ($C_7H_6O_3$, 99%), sodium hypochlorite solution (NaClO, available chlorine 4.0 %), trisodium citrate ($C_6H_5Na_3O_7$, 98%), sodium nitroferricyanide dehydrate ($C_5FeN_6Na_2O \cdot 2H_2O$, 99%), 2-propanol ($C_3H_8O$, 99.5%), N-(1-naphthyl) ethylenediamine dihydrochloride ($C_{12}H_{14}N_2 \cdot 2HCl$, 98%), tetrahydrofuran (C_4H_8O, 99.5%), trimethylamine (C_3H_9N, 30 wt.% in H_2O), and 1-butyl-3-methylimidazolium chloride ($C_8H_{15}ClN_2$, 97%) were purchased from Shanghai Macklin Biochemical Co., Ltd. $CaCO_3$ (99.99% purity, Alfa Aesar), ZnS (99.99% purity, Aladdin), and $MnCO_3$ (>99.99% purity, Sigma-Aldrich) were supplied by the respective commercial sources.

Theoretical calculations

The all-electron exchange-correlation functional was addressed using the generalized gradient approximation (GGA) with the revised Perdew-Burke-Ernzerhof (PBE). To model the catalyst surface, a vacuum layer of at least 15 Å was introduced between periodic slabs to prevent interactions between two periodic units. Brillouin zone sampling was carried out with a 7 × 7 × 5 k-point mesh, and the kinetic energy cutoff was set to 450 eV. The convergence criteria were defined as an energy tolerance of 1.0 $\times 10^{-5}$ Ha, a maximum force of 0.002 Ha/Å, and a self-consistent field (SCF) tolerance of 1.0 $\times 10^{-6}$. The formula for calculating Gibbs Free Energy is as follows:

$$\Delta G = \Delta E_{DFT} + \Delta E_{ZPE} - T\Delta S$$

where ΔE is the electronic energy, ΔZPE is the zero-point energy correction, and ΔS is the entropy contribution. Entropy and zero-point energy corrections were obtained from vibrational frequency calculations and standard thermodynamic data for gas-phase species.

Product quantification

Determination of ammonia: The amount of NH_3 produced was measured using the indophenol blue method. To reduce experimental error in ammonia quantification, each measurement was repeated three times to obtain error bars. Specifically, 1 mL of electrolyte was withdrawn after the NRA test, followed by the addition of 1.25 mL of solution containing 0.625 M NaOH, 0.36 M salicylic acid, and 0.17 M sodium citrate. Subsequently, 150 μL of sodium nitroferricyanide solution (10 mg/mL) and 75 μL of NaClO solution (available chlorine 4.0 wt%) were introduced. After 2 h under ambient conditions, the UV–Vis absorption spectrum was recorded in the range of 550–750 nm, and the absorbance was obtained at 658 nm.

Determination of hydrazine: N_2H_4 in the electrolyte was analyzed using the Watt–Chrisp method. A color reagent was prepared by mixing 50 mL ethanol, 1.0 g para(dimethylamino)benzaldehyde, and 16 mL concentrated HCl. Then, 2 mL of this reagent was added to 2 mL of the electrolyte. After 30 min, the UV–Vis absorption spectrum was recorded between 200 and 300 nm. Standard hydrazine monohydrate solutions with known concentrations in 0.05 M K_2SO_4 were also prepared to construct calibration curves.

Determination of nitrite: NO_2^- was determined using the N-(1-naphthyl) ethylenediamine colorimetric method. The color reagent consisted of 4 g p-aminobenzenesulfonamide, 0.2 g N-(1-naphthyl)ethylenediamine dihydrochloride, 50 mL ultrapure water, and 10 mL phosphoric acid (density 1.70 g/mL). A portion of electrolyte was extracted and diluted to 4 mL for testing. Then, 0.1 mL of the reagent was added, mixed thoroughly, and left to stand for 30 min. The absorbance spectrum was measured at 540 nm using a UV–Vis spectrophotometer to detect the red dye. The final absorbance of NO_2^- was calculated at 540 nm. The standard calibration curve was established by the absorbance of the given $NaNO_2$.

Determination of gas-phase products. The gas products and Ar feeding gas were collected with a gas sampling bag and further analyzed through a gas chromatograph (GC) (Model 450-GC, Bruker) equipped with TCD.

Faradaic efficiency and ammonia yield rate

FEs and area-normalized yield rates of NH_3 and N_2H_4 were calculated:

FE (NH_3) = $(8F \times C \times V) / Q$

Yield rate (NH_3) = $(17C \times V) / (t \times A)$

FE (N_2H_4) = $(7F \times C \times V) / Q$

Yield rate (N_2H_4) = $(32C \times V) / (t \times A)$

Where F is the Faraday constant (96485 C/mol), C is the measured NH_3 concentration, V is the volume of electrolyte, Q is the total charge passed through the electrode, A is the geometer area of the working electrode.

Determination of nitrate. First, a 5-mL solution was taken out from the anodic cell. Then 0.10 mL of 1.0 M HCl solution was added into the above solution. After shaking for 10 min and standing for 5 min, the absorbance of NO_3^- was measured by using an UV-Vis spectrophotometer at a wavelength range from 300 nm to 200 nm. The final absorbance (**A**) of NO_3^- was calculated based on the equation: $A = A_{220nm} - 2A_{275nm}$. The standard calibration curve was established by the absorbance of the different given concentrations of $NaNO_3$.

Faradaic efficiency and yield rate of nitrite

FEs and area-normalized yield rates of NO_2^- were calculated:

FE (NO_2^-) = $(2F \times C \times V) / Q$

Yield rate (NO_2^-) = $(C \times V) / (t \times A)$

Where F is the Faraday constant (96485 C/mol), C, V, Q, and A are the measured NO_2^- concentration, the volume of electrolyte and the total charge passed through the electrode and the geometer area of the working electrode.

^{15}N isotope labeling experiment: $K^{15}NO_3$ with an isotope abundance of 99% was used as a nitrogen source in isotope-labeled nitrate reduction experiments to determine the source of ammonia. 0.1 M $K^{15}NO_3$ was added to PBS solution as the electrolyte. After the NRA test, samples were extracted from the electrolyte containing generated $^{15}NH_4^+$, mixed with 0.05-mL deuterium oxide (D_2O), and further qualitative analysis was conducted via 600 MHz 1H NMR.

Hume–Rothery rules

$$D_r = 100\% \times \frac{R_m(\mathrm{CN}) - R_d(\mathrm{CN})}{R_m(\mathrm{CN})}$$

where D_r, CN, R_m(CN), and R_d(CN) represents the percentage difference in ionic radii, the coordination number, the radius of the host cation, and the radius of the dopant ion, respectively.

S2. Figures and tables

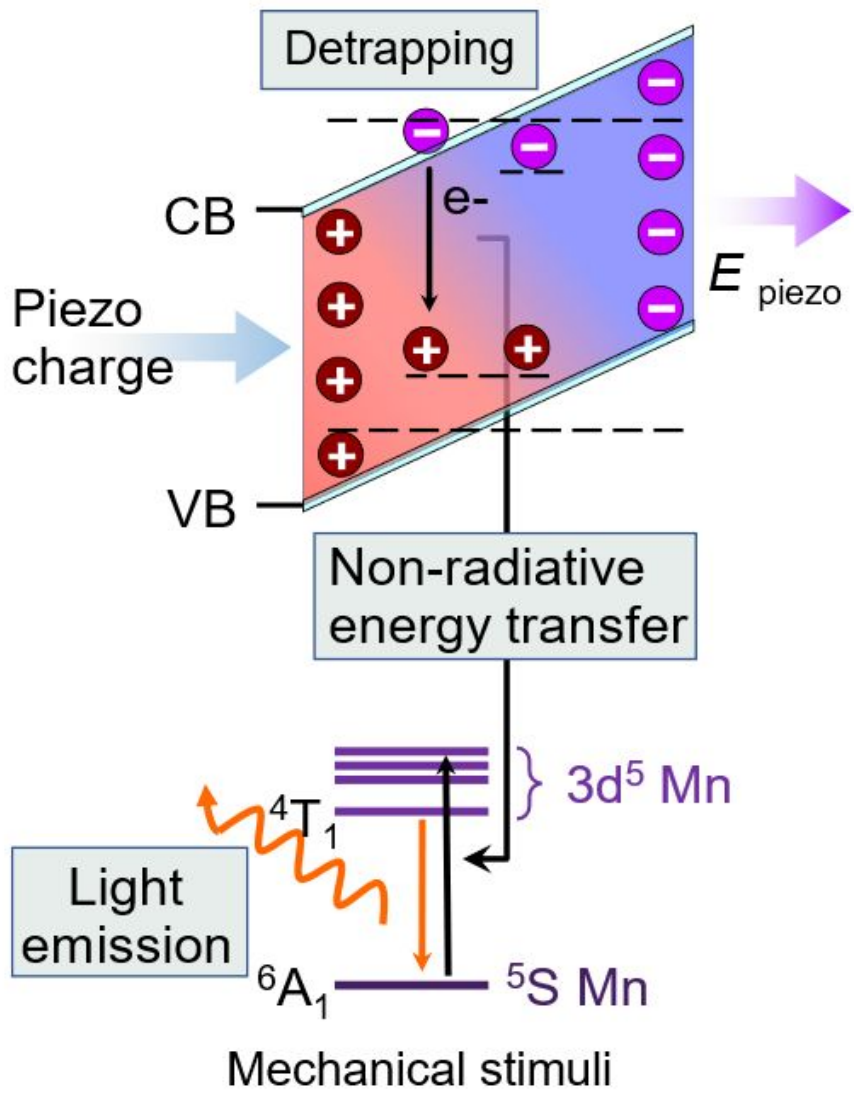

Figure S1. The reported mechanism of piezophotonic effect for the ML process (*Adv. Mater.*, 2025, 27, 2324-2331).

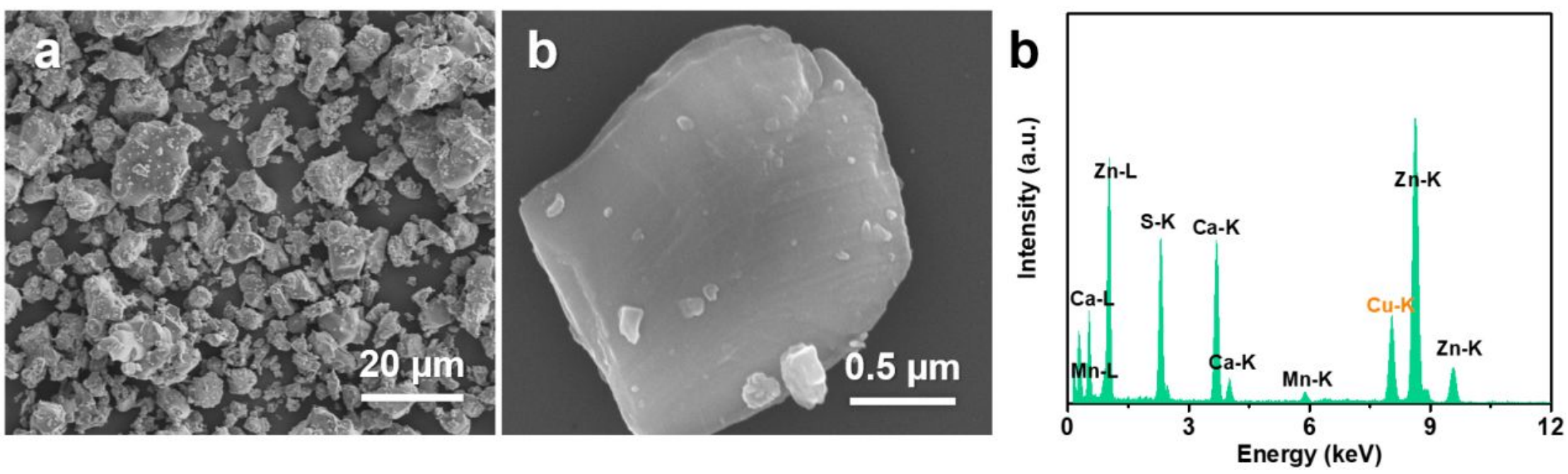

Figure S2. (a-b) SEM images and EDX spectrum of Mn-doped CaZnOS. The Cu signal is attributed to the Cu substrate.

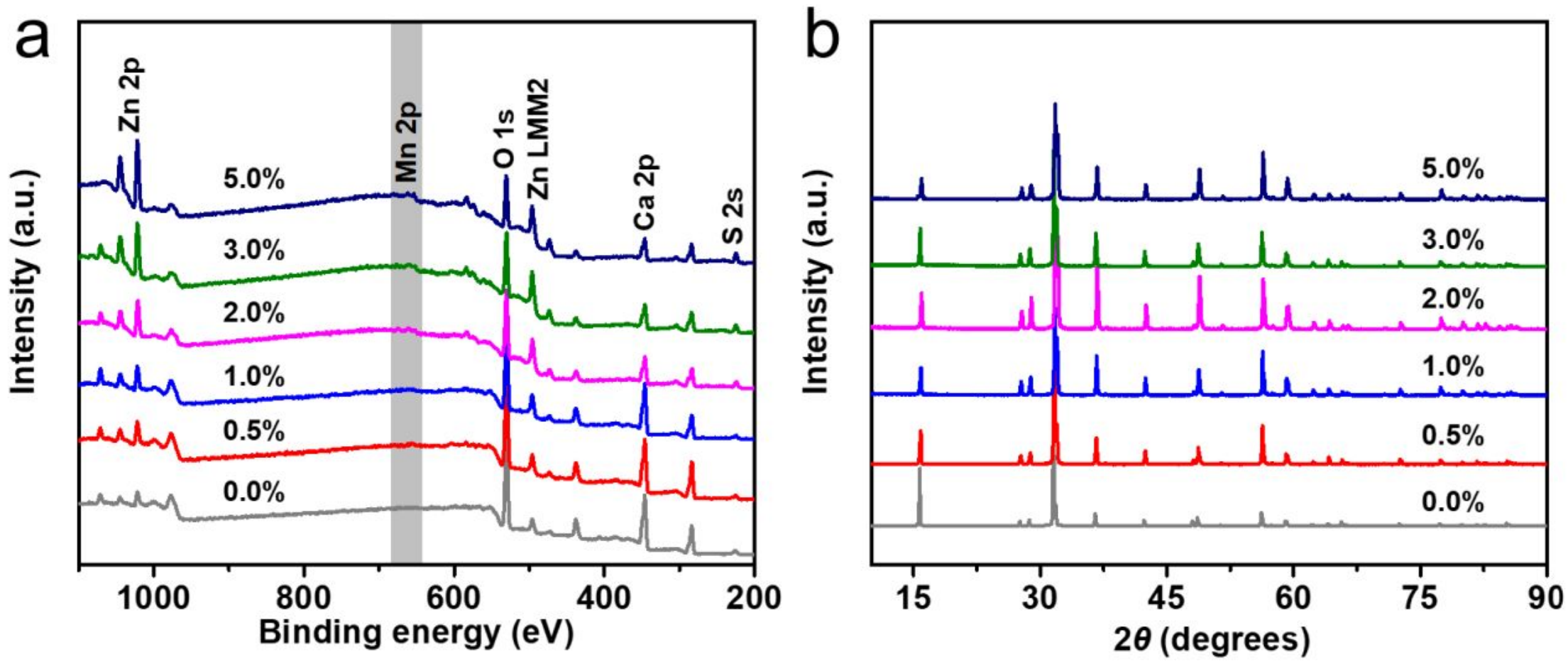

Figure S3. (a) Survey XPS spectra and (b) XRD patterns of the as-prepared Mn-CaZnOS samples with different weight ratios of the Mn content.

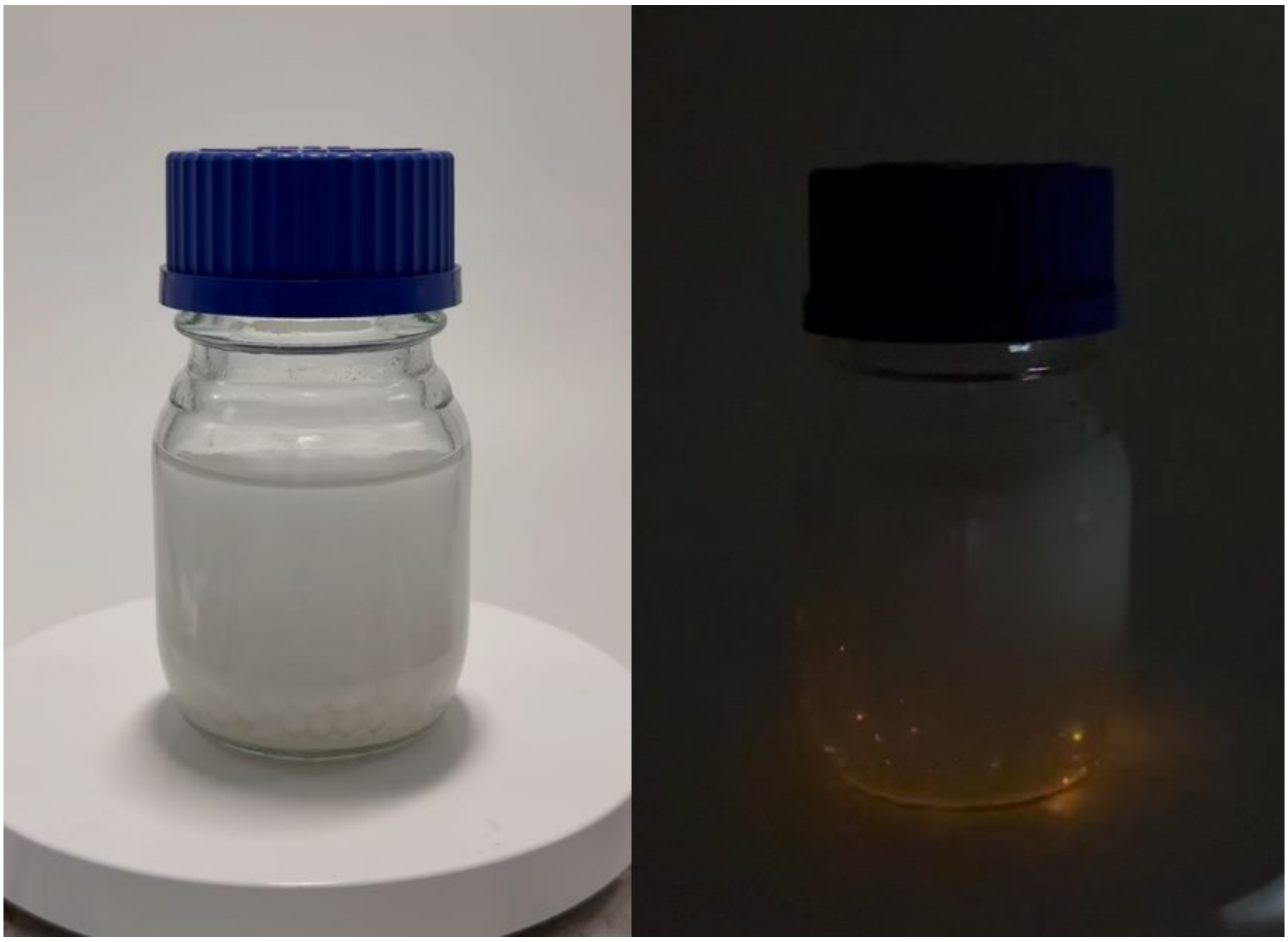

Figure S4. Real photos of the ML-enhanced NRA conversion over the Mn-CaZnOS catalyst.

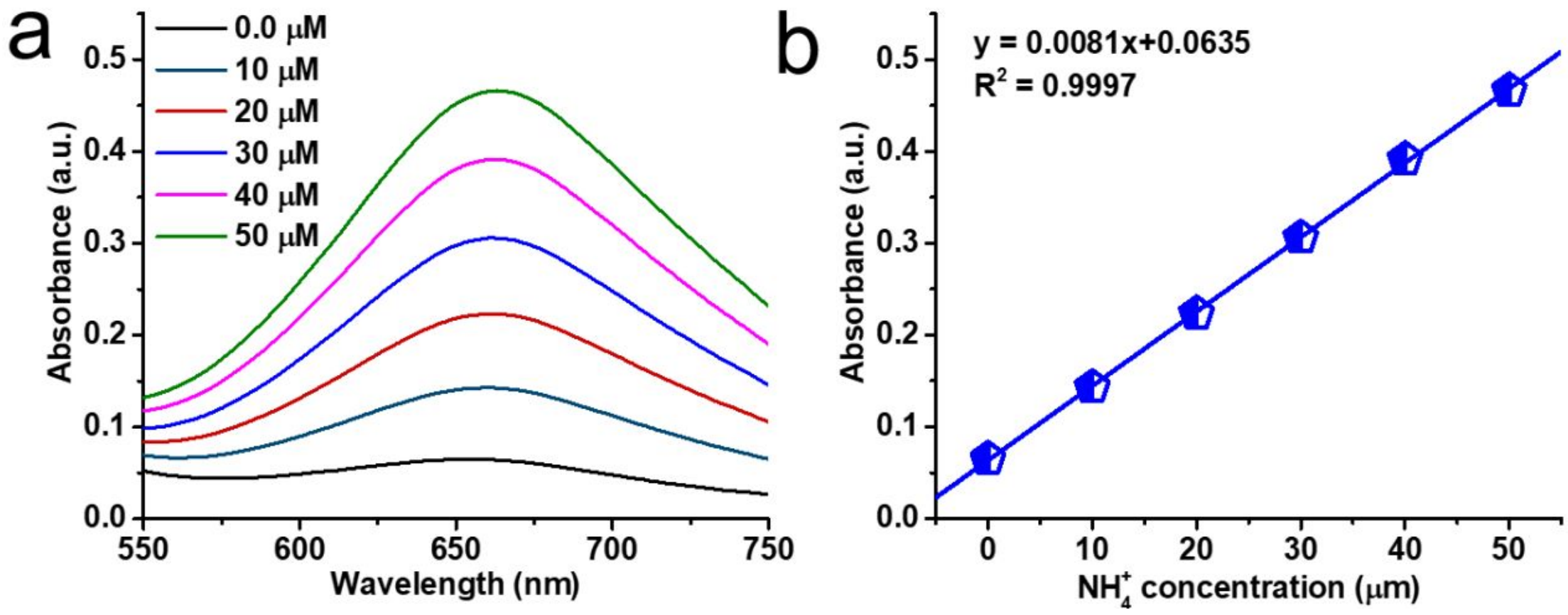

Figure S5. The UV-Vis curves and calibration curves of the DI water electrolyte with the given concentrations of NH_3.

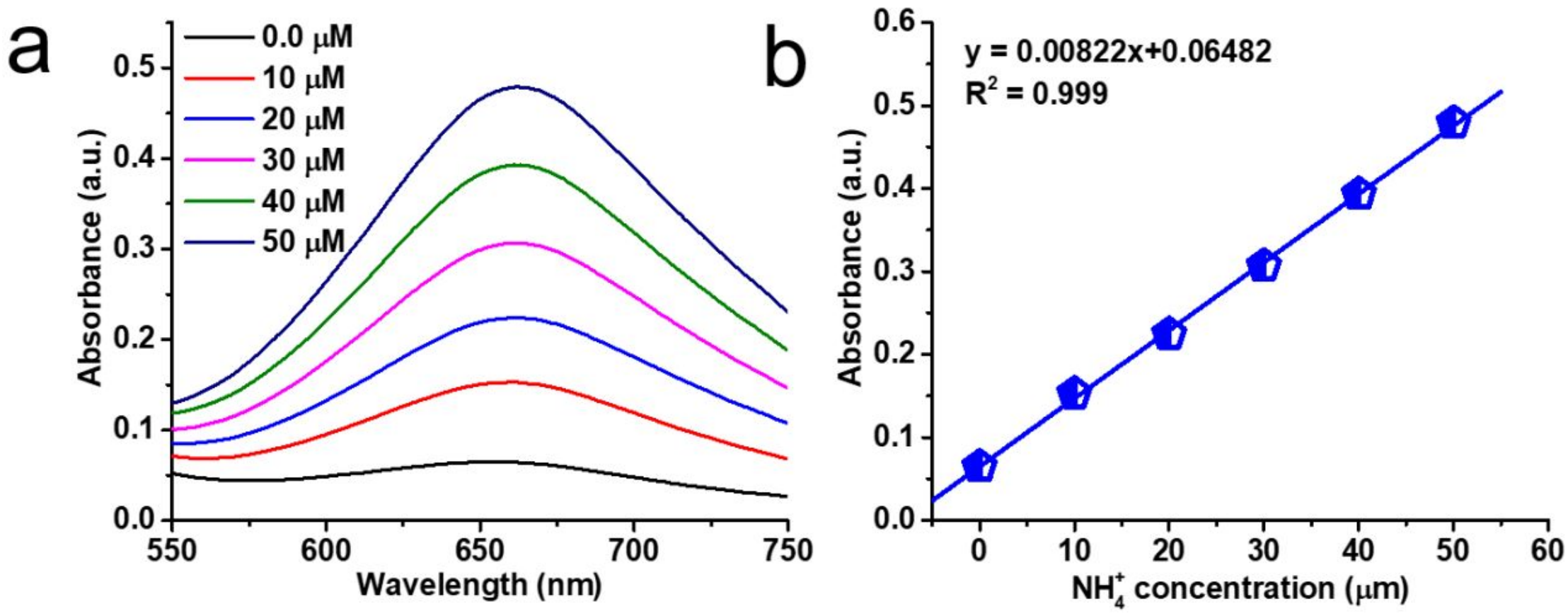

Figure S6. The UV-Vis curves and calibration curves of the PBS electrolyte with the given concentrations of NH_3.

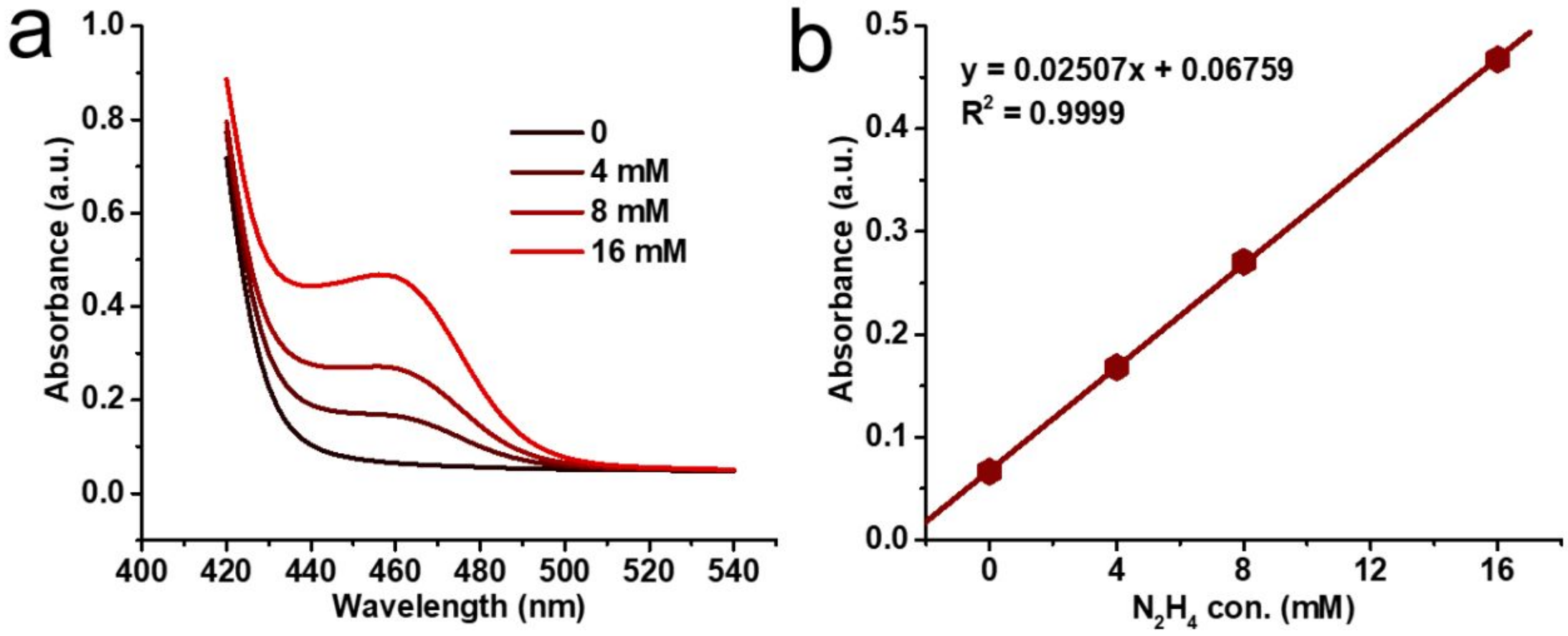

Figure S7. The UV-Vis curves and calibration curves of the PBS electrolyte with the given hydrazine concentrations.

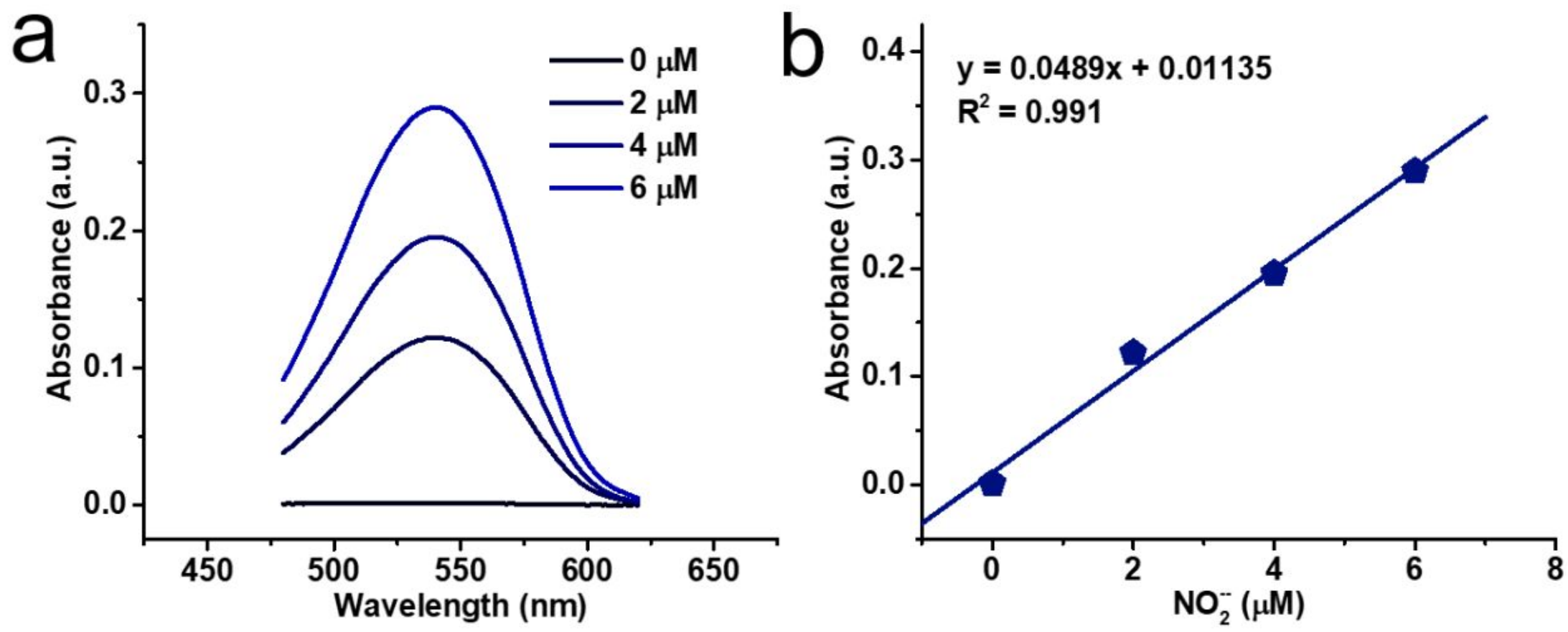

Figure S8. The UV-Vis curves and calibration curves of the PBS electrolyte with the given NO_2^- concentrations.

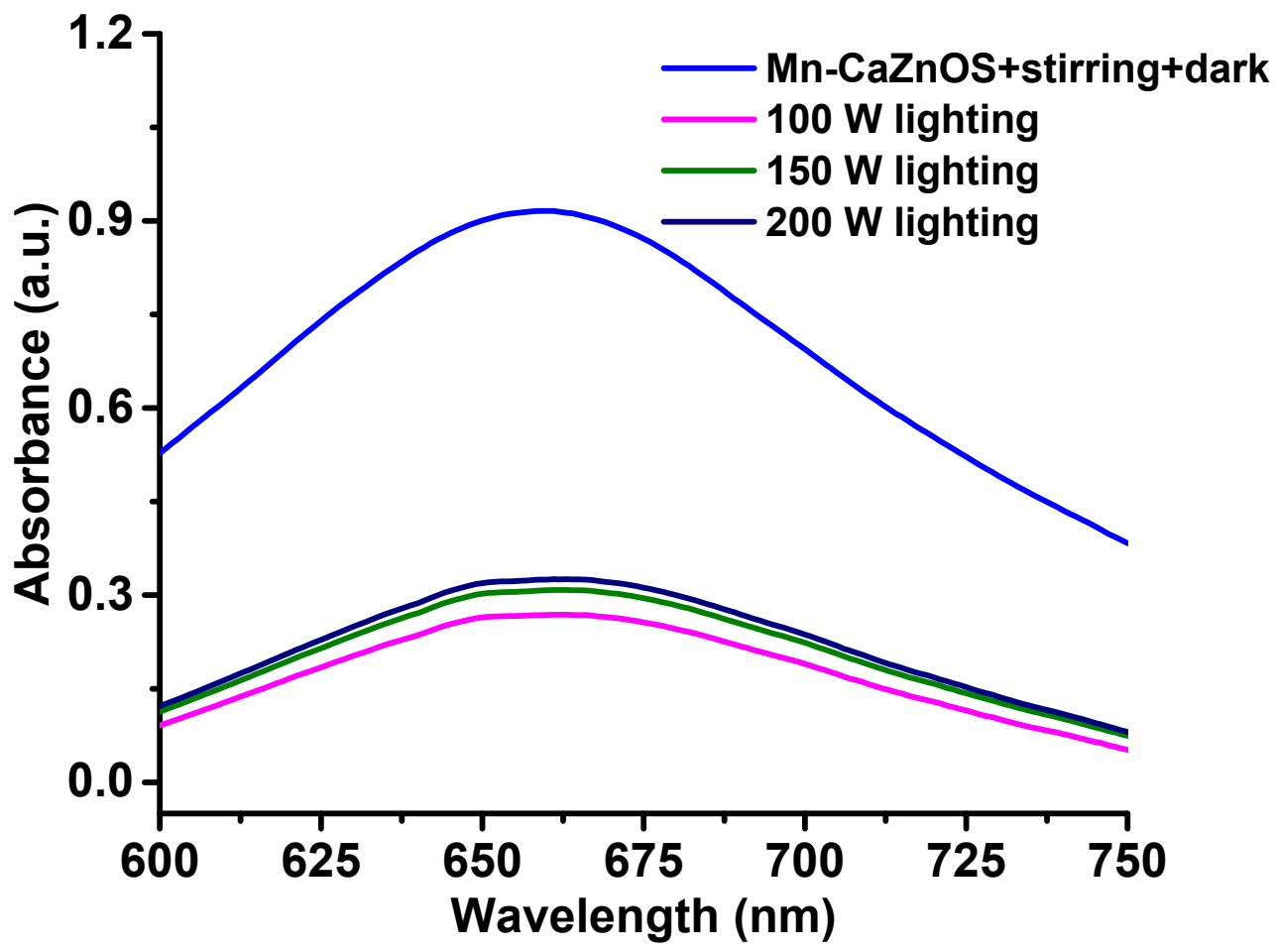

Figure S9. UV-vis spectra of the electrolytes containing NH_3 achieved with 500 mg of Mn-CaZnOS and 0.1 M NO_3^- under experimental conditions using DI water as the electrolyte.

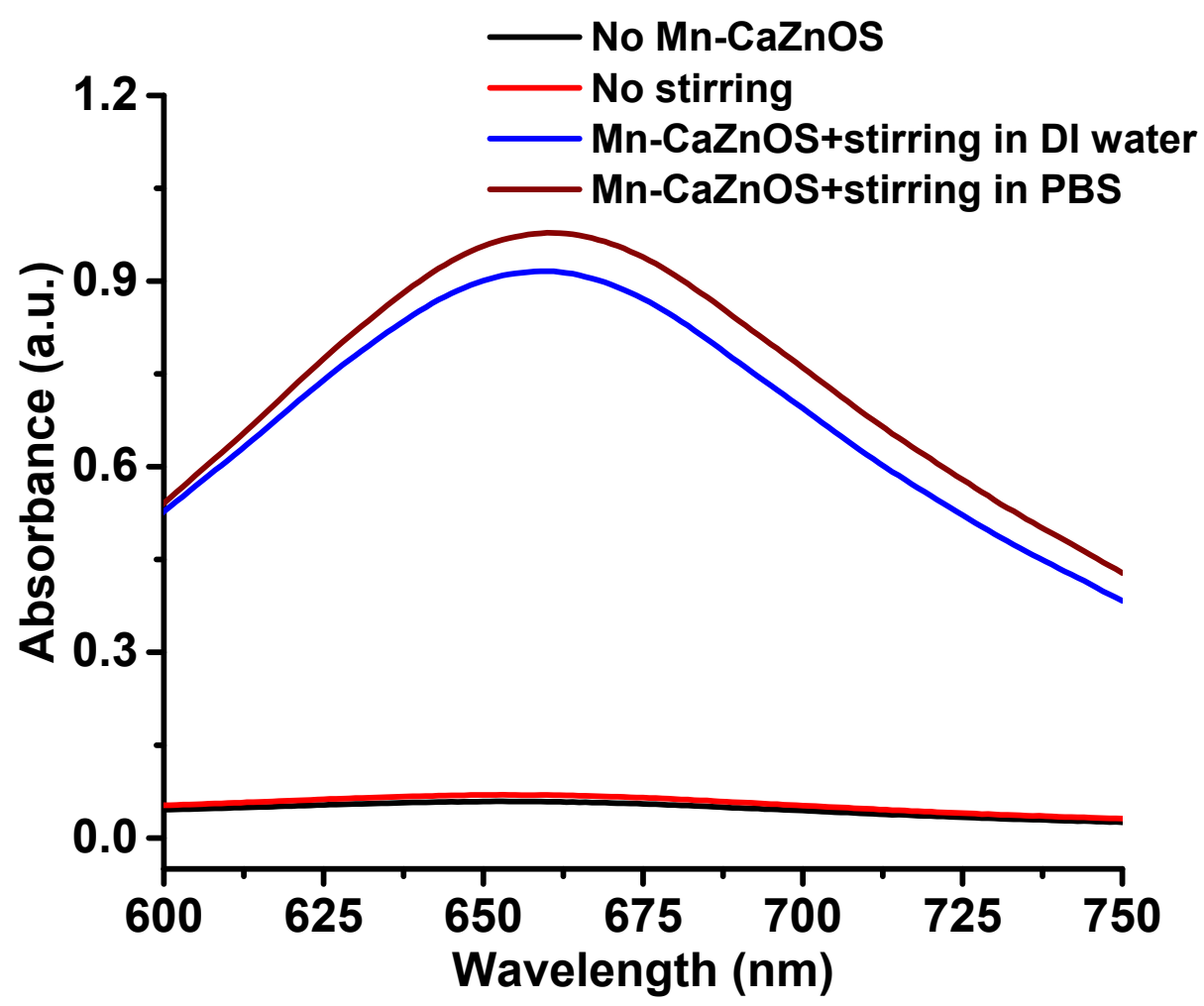

Figure S10. UV-vis spectra of the electrolytes containing NH_3 achieved with 500 mg of Mn-CaZnOS and 0.1 M NO_3^- under different conditions using DI water or PBS as the electrolyte in the dark environment.

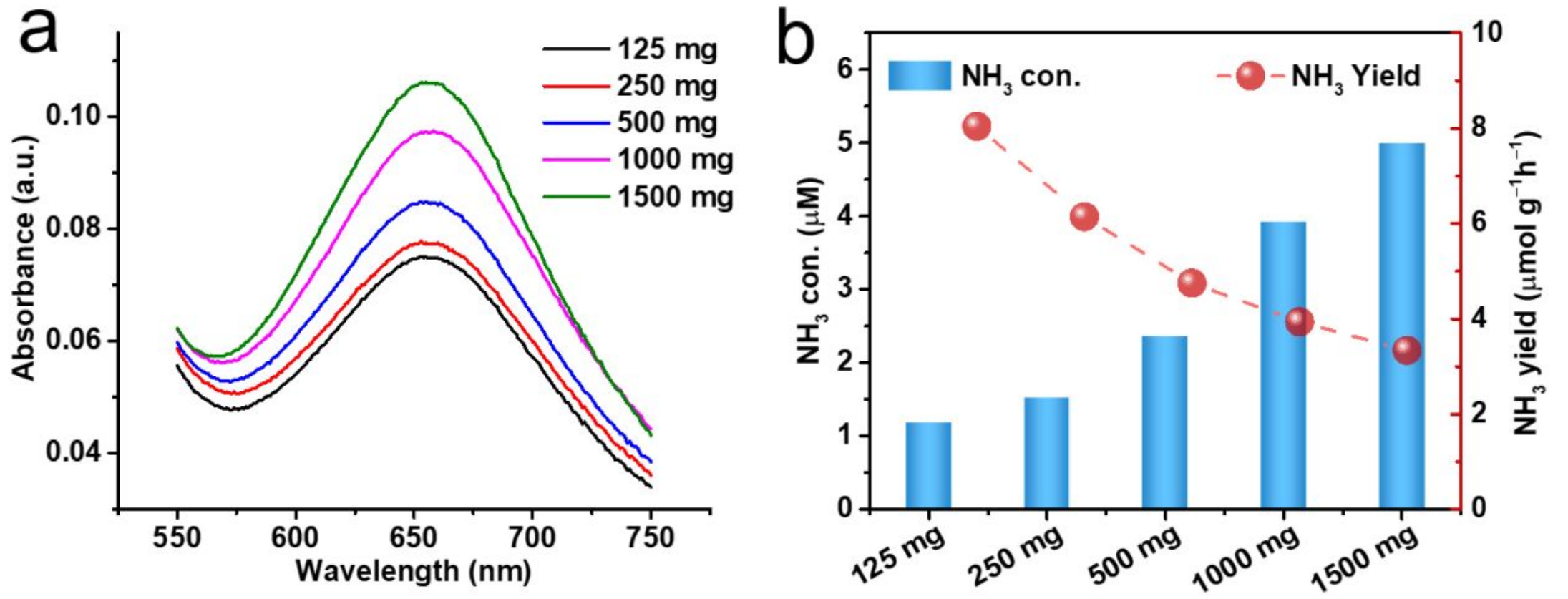

Figure S11. (a) The UV-Vis curves and (b) NH_3 production of the pristine CaZnOS catalyst using the PBS electrolyte.

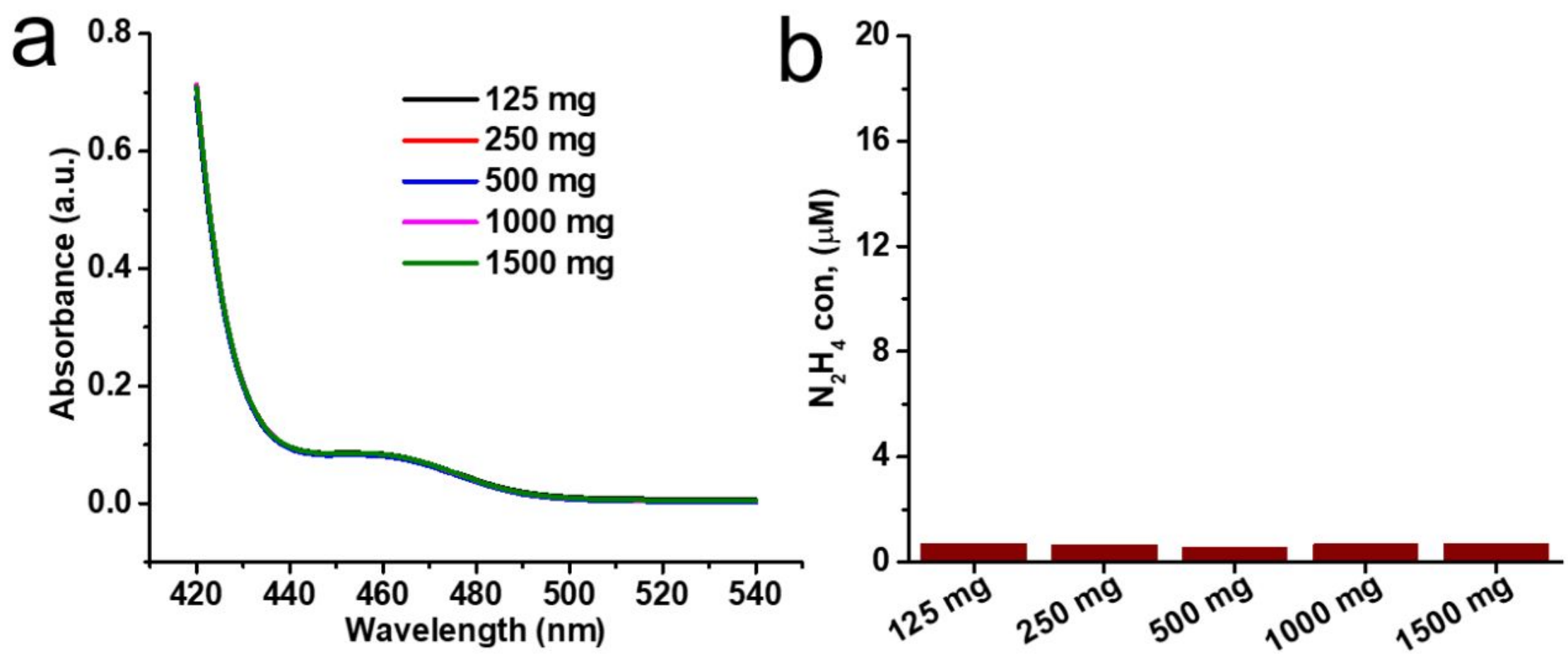

Figure S12. (a) The UV-Vis curves and (b) N_2H_4 of the resultant electrolyte.

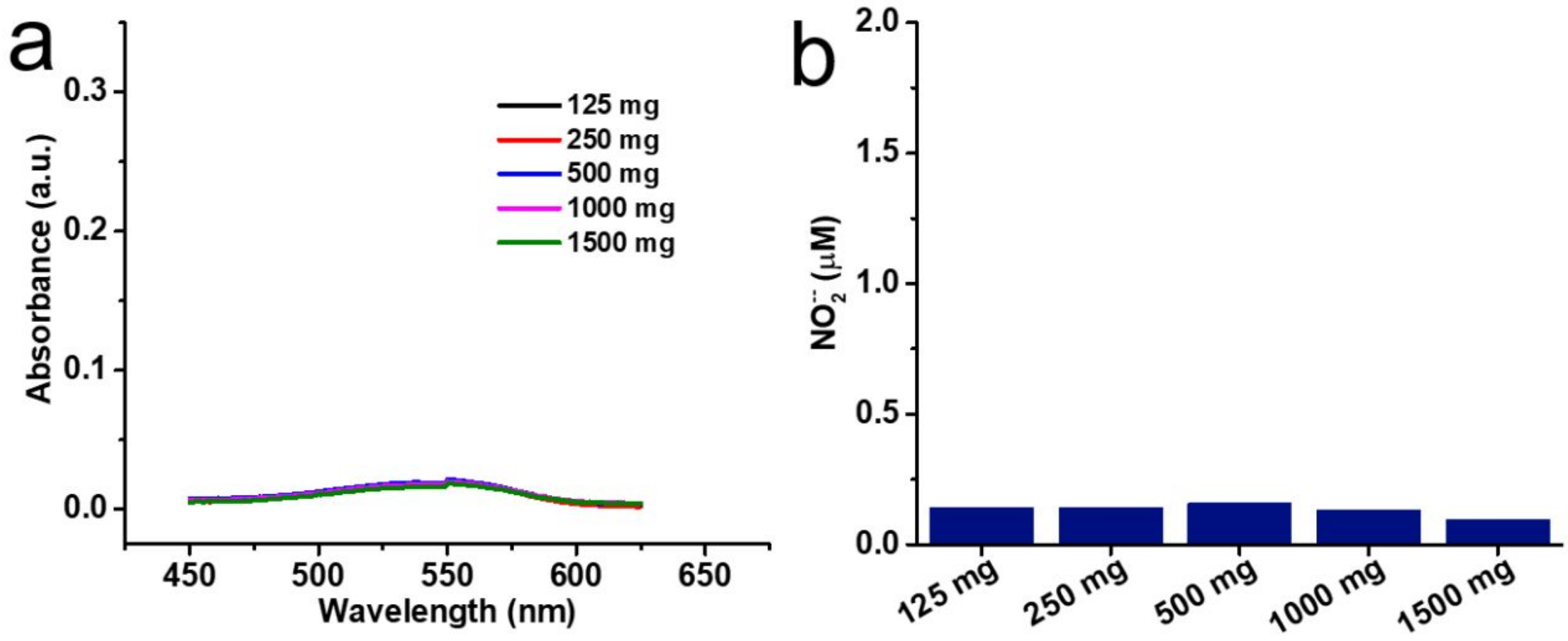

Figure S13. (a) The UV-Vis curves and (b) NO_2^- of the resultant electrolyte.

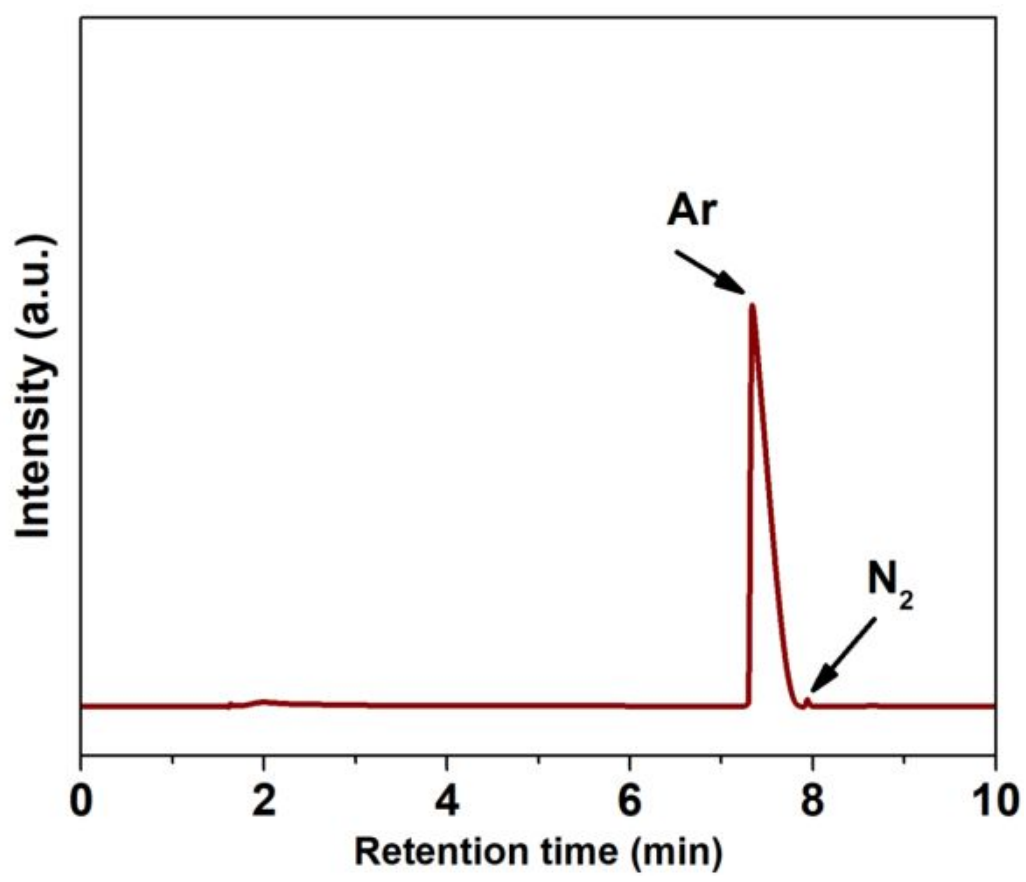

Figure S14. The representative GC-TCD chromatogram of the gas product reveals a main peak signal of Ar feeding gas and a quite tiny peak of N_2.

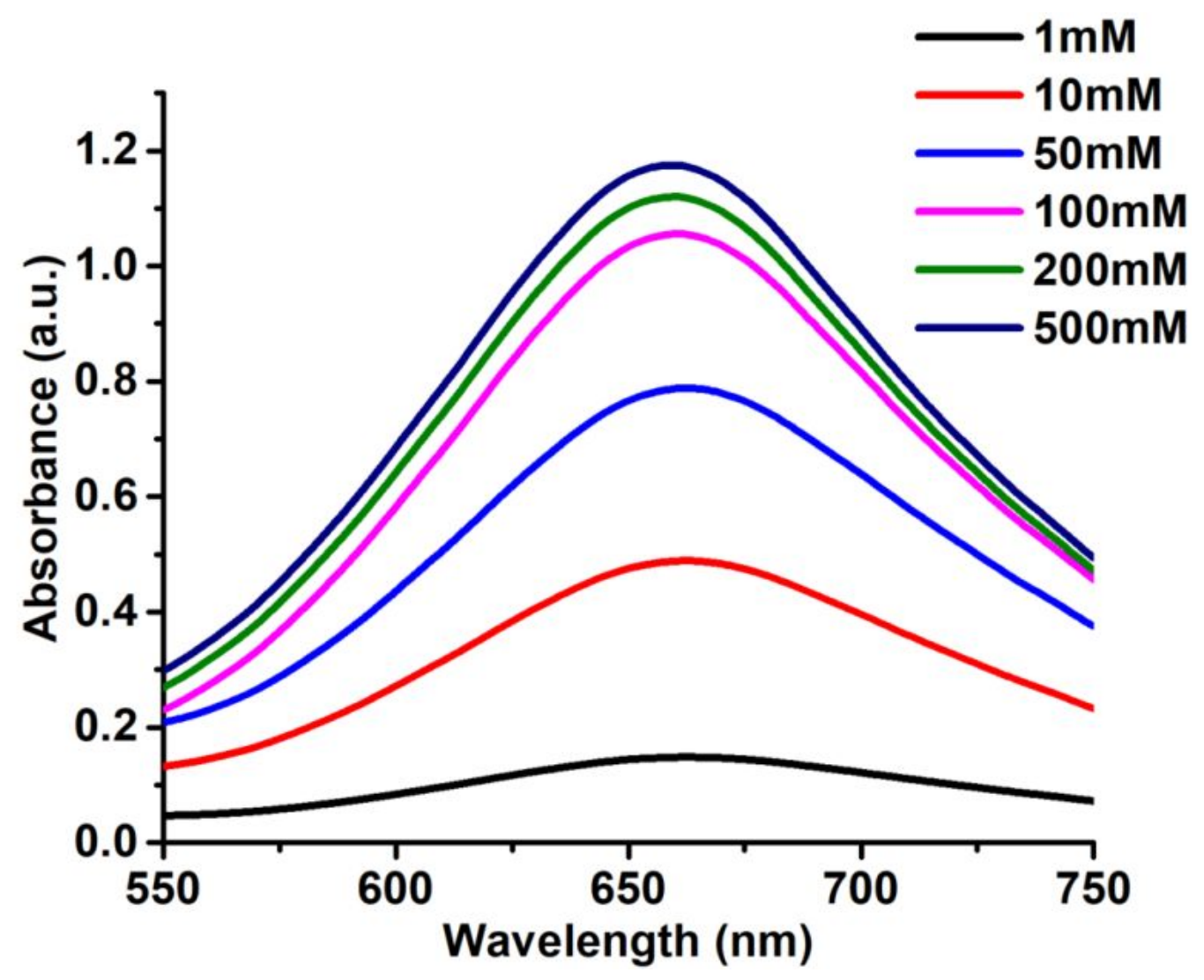

Figure S15. UV-vis spectra of the PBS electrolytes containing NH_3 achieved with 1000 mg of Mn-CaZnOS and different NO_3^- concentration in the dark environment.

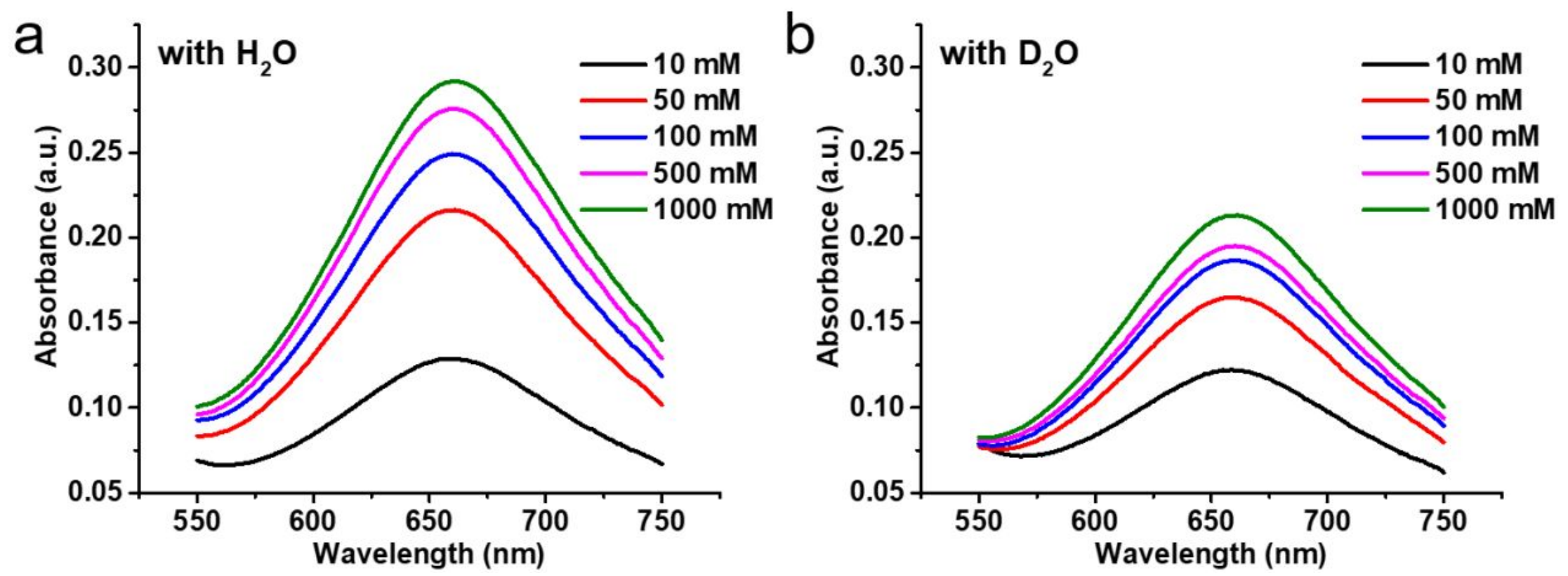

Figure S16. UV-vis spectra of the resultant PBS electrolytes using H_2O and D_2O as solvent in the dark environment.

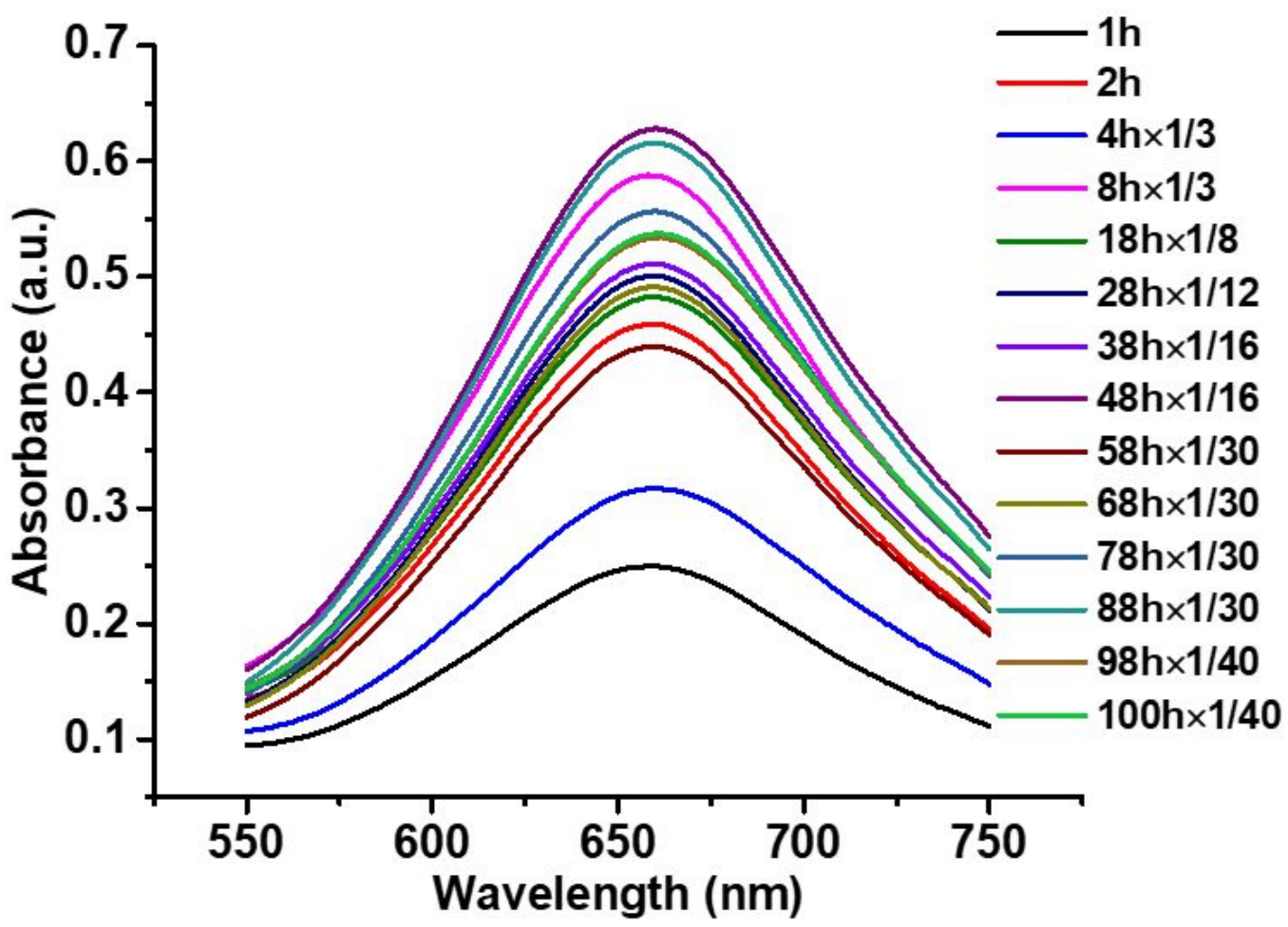

Figure S17. UV-vis spectra of the resultant PBS electrolytes after the long-term test in the dark environment.

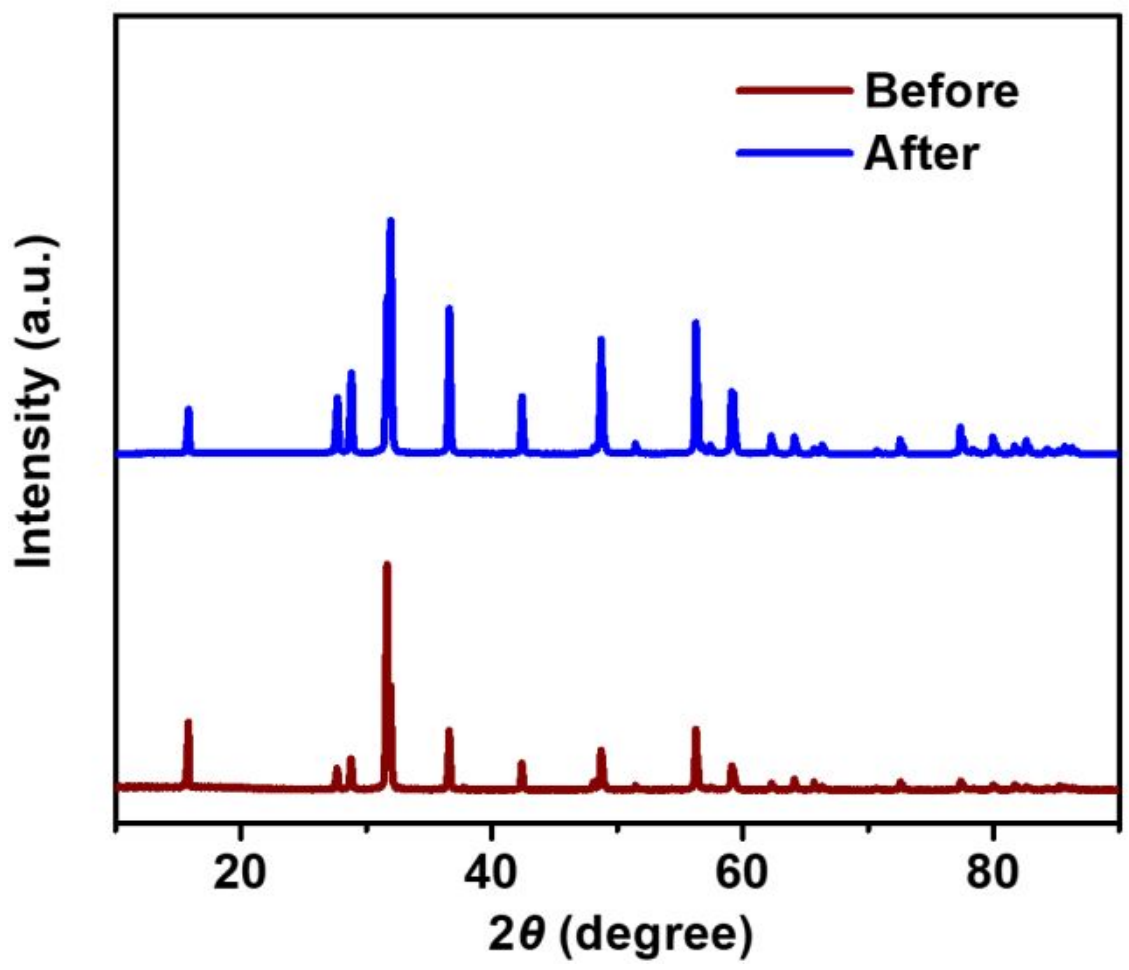

Figure S18. The XRD patterns of the Mn-CaZnOS sample before and after the long-term NRA test.

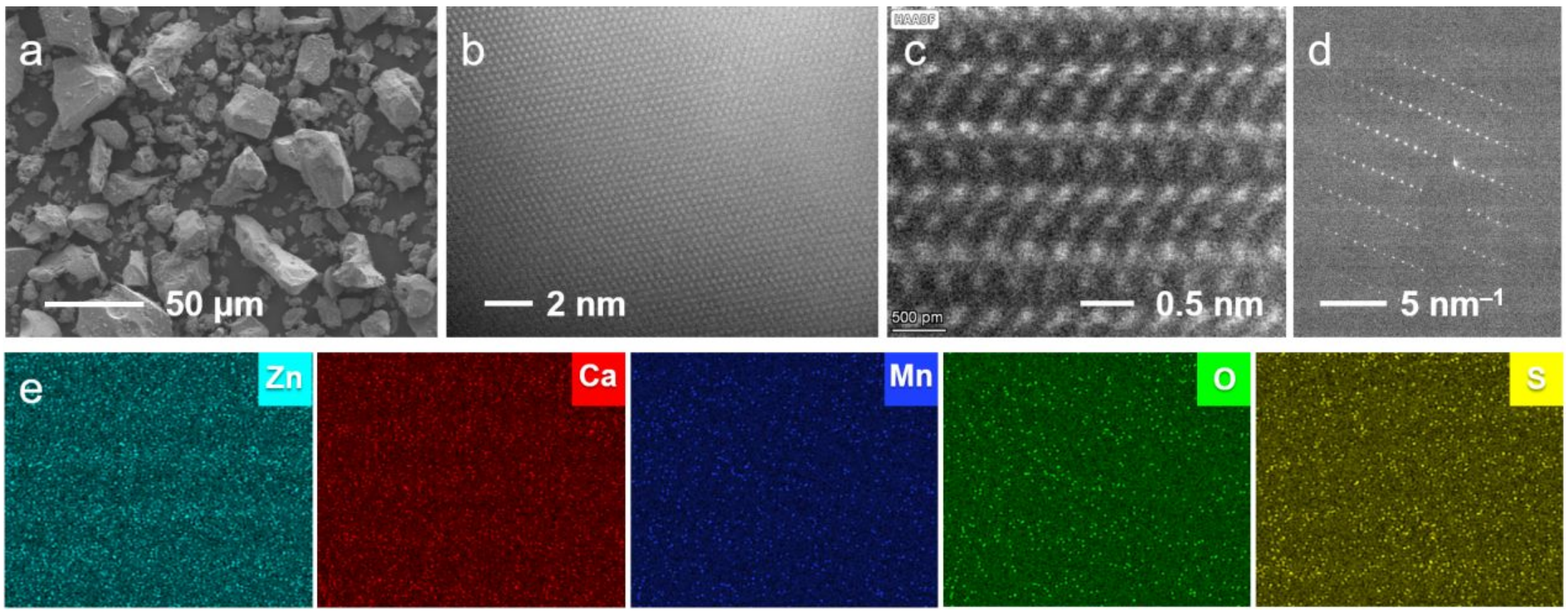

Figure S19. (a) SEM image, (b, c) HAADF-STEM images, and (d) the FTT pattern of Mn-CaZnOS after the long-term NRA test.

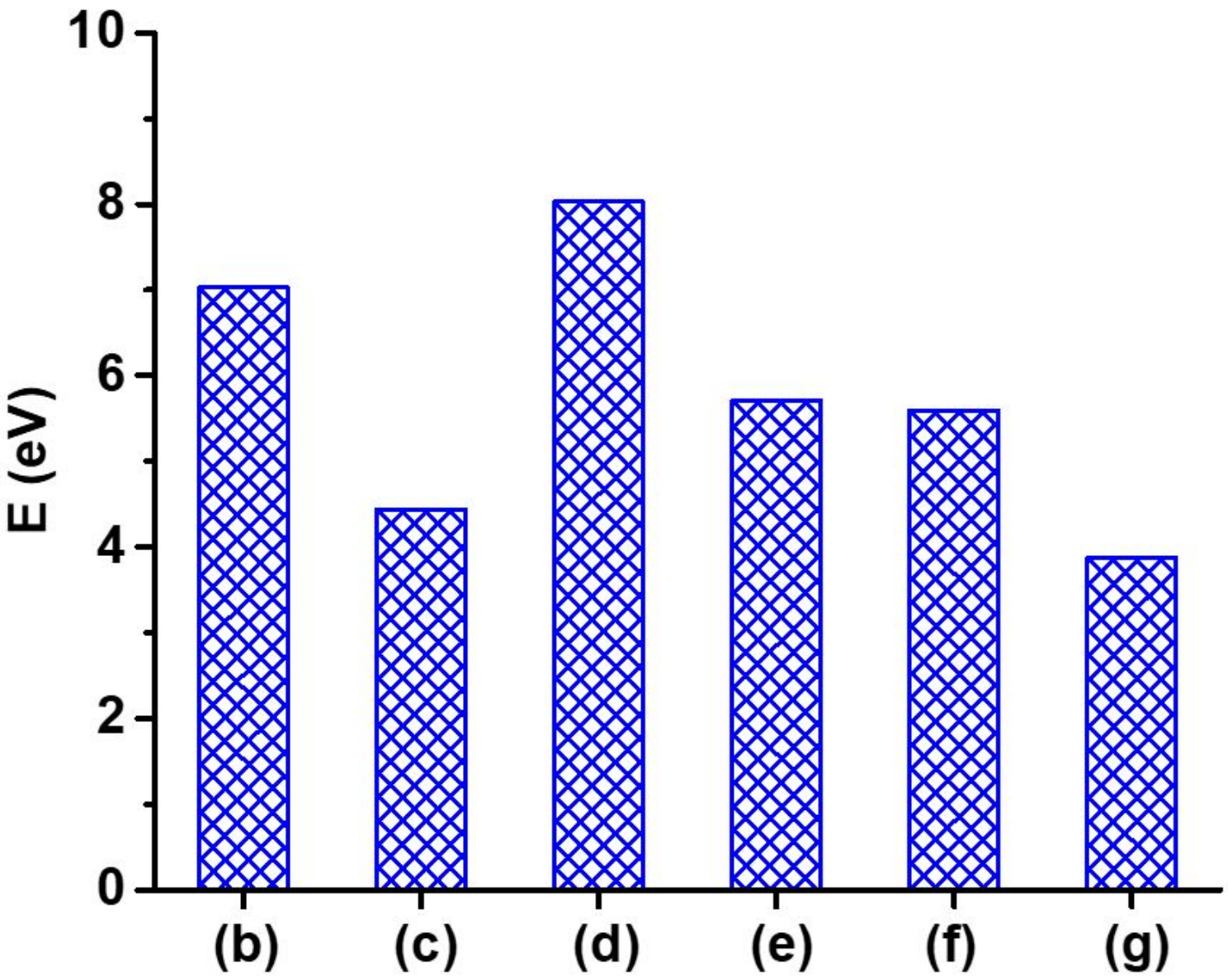

Figure S20. The total energy of seven optimized models derived from **Table S1**, indicating that the S vacancy in Mn-doped CaZnOS with the substitution of Mn to Ca (Mn-CaZnOS-Ca-Vs).

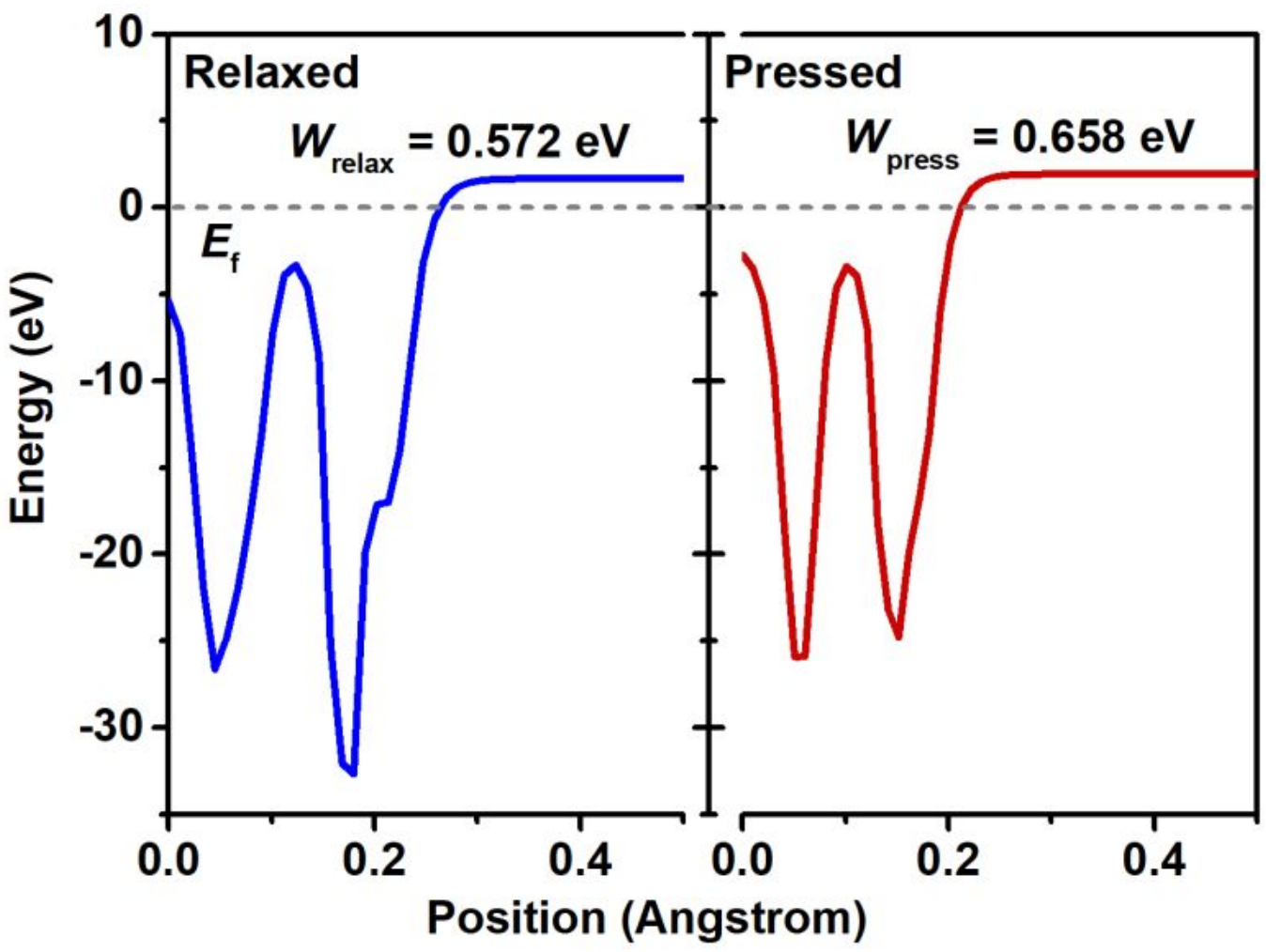

Figure S21. Work functions of the relaxed and pressed Mn-CaZnOS models.

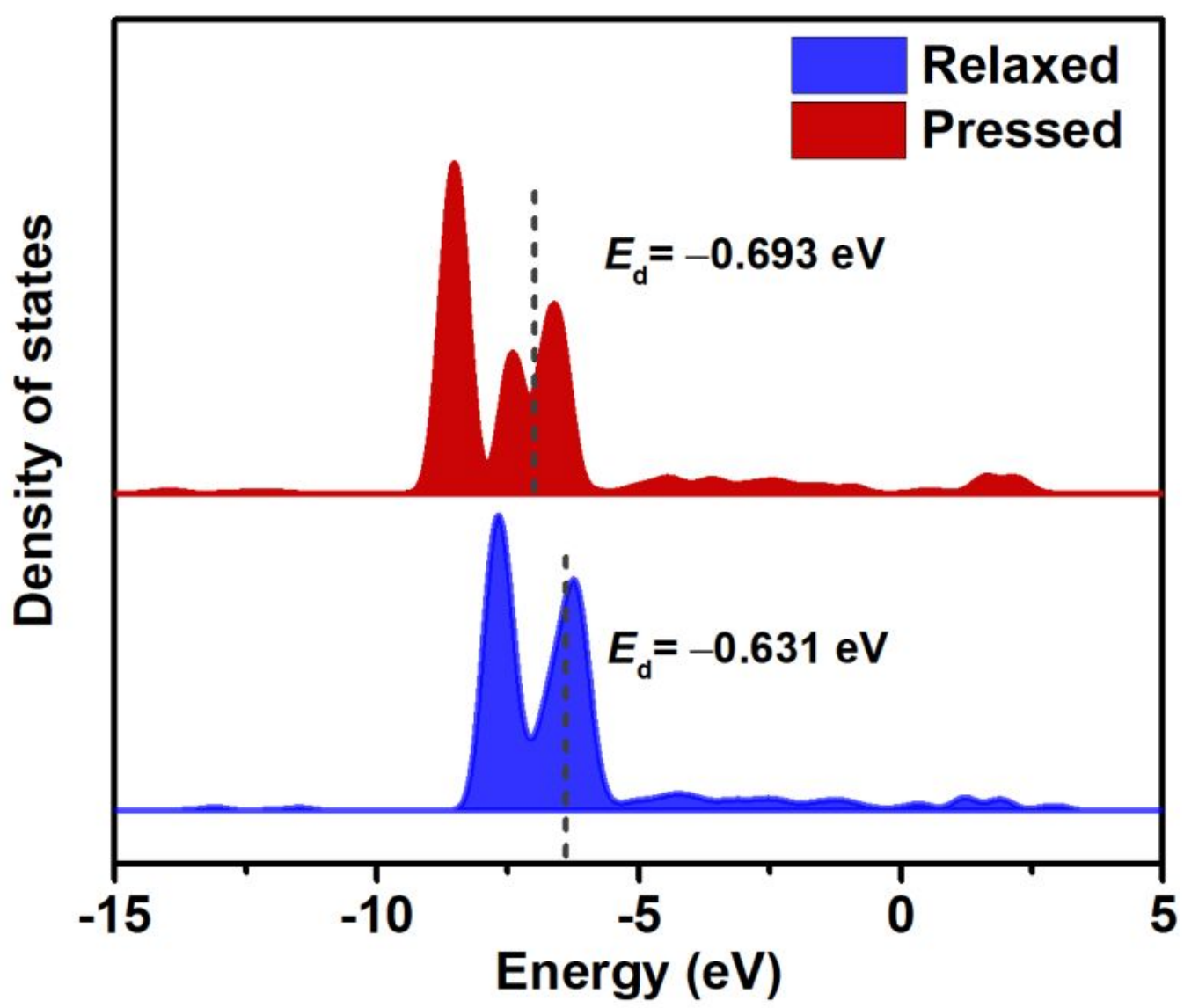

Figure S22. Investigation of d band centers of the relaxed and pressed Mn-CaZnOS catalyst.

Table S1. Mn wt% determined from ICP-MS test.

**Sample**	**Mn wt%**
0.5%Mn-CaZnOS	0.462
1.0%Mn-CaZnOS	1.086
2.0%Mn-CaZnOS	1.865
3.0%Mn-CaZnOS	2.958
5.0%Mn-CaZnOS	4.543

Table S2. Performance comparison of the previously reported systems for nitrate reduction to ammonia.

Catalysis type	Catalyst	NH_3 yield	Reference
	CoNi-Vp-1.0	12.2 mmol g^{-1} h^{-1}	Appl. Catal. B-Environ. 330, 122627 (2023).
	Pd/TiO_2	3.1 mmol g^{-1} h^{-1}	nergy Environ. Sci. 14, 3938-3944 (2021).
	Fe/Ni_2P	22.3 mmol g^{-1} h^{-1}	Adv. Energy Mater. 12, 2103872 (2022).
	i-Ag/Co_3O_4 NWs	42.7 mmol g^{-1} h^{-1}	Adv. Sci. 10, 2303789 (2023)
	$Co_2B@Co_3O_4$/TM	43.5 mmol g^{-1} h^{-1}	ACS Appl. Mater. Interfaces 14, 49650-49657 (2022).
	Co_2AlO_4	39.7 mmol g^{-1} h^{-1}	Chem. Eng. J. 435, 135104 (2022).
	PdCuAg MTs	25.8 mmol g^{-1} h^{-1}	Adv. Energy Mater. 13, 2303054 (2023).
	$NiCo_2O_4$	48.2 mmol g^{-1} h^{-1}	Small 18, 2106961 (2022).
	Fe_2TiO_5	32.3 mmol g^{-1} h^{-1}	Angew. Chem. Int. Ed. 135, e202215782 (2023).
	Ir SAC-Co_3O_4	44.9 mmol g^{-1} h^{-1}	Chem Catal. 3, 100477 (2023).
	2D Cu plate	52.3 mmol g^{-1} h^{-1}	Angew. Chem. Int. Ed. 136, e202401924 (2024).
Photocatalysis	NH_2-MIL-125/$Co(OH)_2$	2.45 mmol g^{-1} h^{-1}	Angew. Chem. Int. Ed. 63, e202409163 (2024).
	CuO_x:TiO_2	1.64 mmol g^{-1} h^{-1}	ACS Appl. Energy Mater. 7, 6527-6536 (2024).
	Pt SAs/$Ti_{0.87}O_2$ NSs	35.65 µmol g^{-1} h^{-1}	J. Am. Chem. Soc. 147, 9049-9055 (2025).
	$ZnIn_2S_4$-$CuIn_5S_8$	117.32 µmol g^{-1} h^{-1}	Appl. Catal. B-Environ. Energy 383, 126110 (2026).
	Pt SAs/p-TiO_2 NTs	21.87 µmol g^{-1} h^{-1}	Angew. Chem. Int. Ed. 137, e202415865 (2025).
	Au_2-COF	382.48 µmol g^{-1} h^{-1}	Chinese J. Catal. 73, 358-367 (2025).
	Cu(6)-TiO_2_650	0.93 µmol g–1 h–1	J. Am. Chem. Soc. 147, 1968-1979 (2025).
	NVCN475	8.83 µmol g^{-1} h^{-1}	Chem. Eng. J. 484, 149506 (2024).
	Ni/H_xWO_{3-y}	10.5 mmol g^{-1} h^{-1}	Appl. Catal. B-Environ. 341, 123266 (2024).
	Cu-NM	1.93 mmol g^{-1} h^{-1}	Nano Energy 124, 109499 (2024).

Piezo-photocatalysis	$CdS@SnS_2$	8 mmol g^{-1} h^{-1}	Compos. Part B-Eng. 293, 112108 (2025)
	N-PDA	5.36 mmol g^{-1} h^{-1}	Appl. Catal. B-Environ. Energy 361, 124558 (2025).
	ZnO@ZnSe	2.88 mmol g^{-1} h^{-1}	Adv. Sci. 13, e18794 (2026).
	C_3N_4/Cu	1.48 mmol g^{-1} h^{-1}	ACS Catal. 15, 4025-4038 (2025).
Mechano-catalysis	Mn-CaZnOS	5.4 µmol g^{-1} h^{-1}	This work

As shown in **Table S2**, although the NH_3 yield achieved with the Mn-CaZnOS mechanocatalyst is lower than those reported for electrocatalytic, piezo-photocatalytic, piezo-catalytic, and photocatalytic systems, ML-enhanced NH_3 production via mechanochemical nitrate reduction remains scarcely explored. This work therefore introduces a novel design principle for mechanically driven catalytic systems: leveraging ML to activate reaction pathways without relying on external light or electrical inputs. By decoupling ammonia synthesis from continuous external energy sources, the approach offers clear advantages for off-grid and distributed deployment and warrants further optimization to improve catalytic performance and scalability.

Table S3. Top view and side view of the optimized model structures. (a) the original CaZnOS with the (100) facet (CZOS), (b) Mn-doped CaZnOS with the substitution of Mn to Zn (Mn-CaZnOS-Zn), (c) Mn-doped CaZnOS with the substitution of Mn to Ca (Mn-CaZnOS-Ca), (d) the O vacancy in Mn-CaZnOS-Zn (Mn-CaZnOS-Zn-Vo), (e) the O vacancy in Mn-CaZnOS-Ca (Mn-CaZnOS-Ca-Vo), (f) the S vacancy in Mn-CaZnOS-Zn (Mn-CaZnOS-Zn-Vs), and (g) the S vacancy in Mn-CaZnOS-Ca (Mn-CaZnOS-Ca-Vs).

(a)	(b)	(c)	(d)	(e)	(f)	(g)
CZOS	Mn-CaZnOS-Zn	Mn-CaZnOS-Ca	Mn-CaZnOS-Zn-Vo	Mn-CaZnOS-Ca-Vo	Mn-CaZnOS-Zn-Vs	Mn-CaZnOS-Ca-Vs
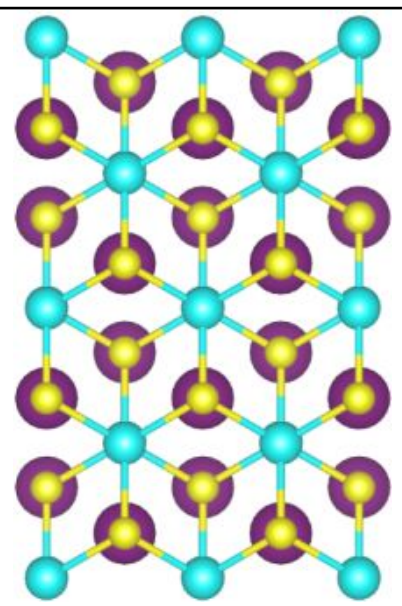	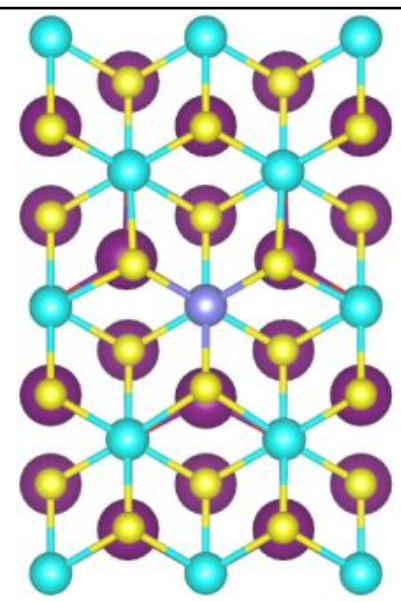	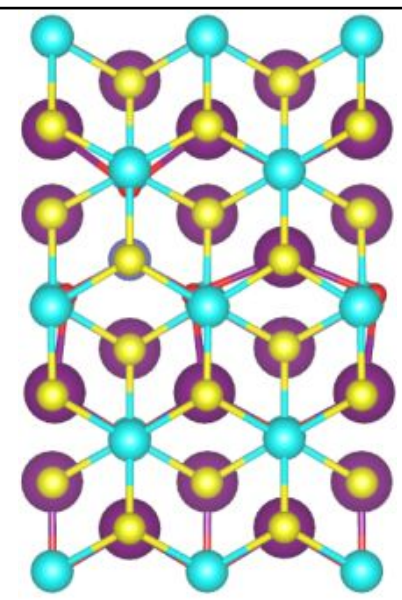	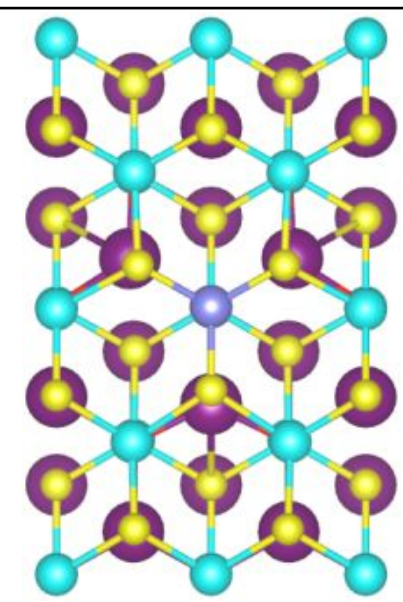	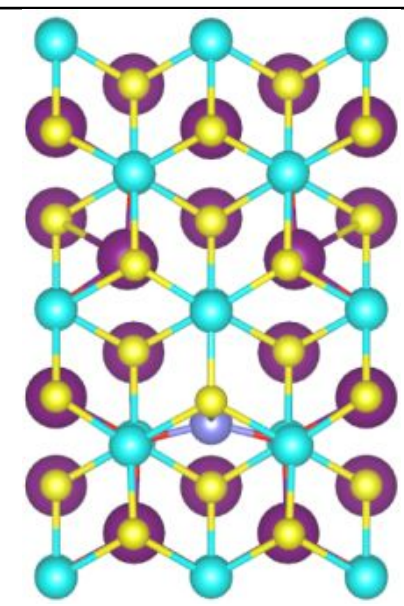	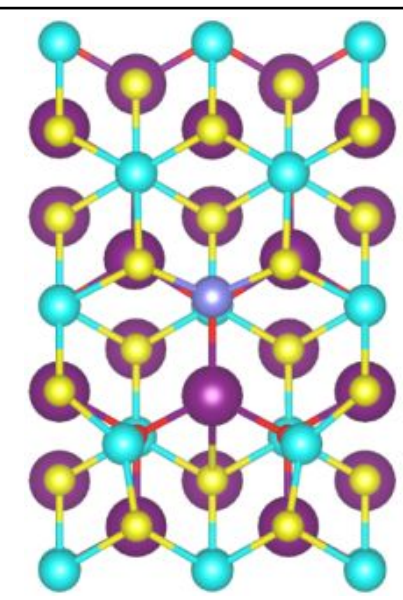	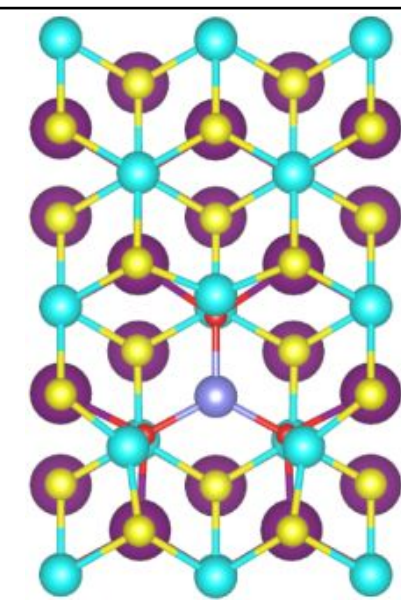
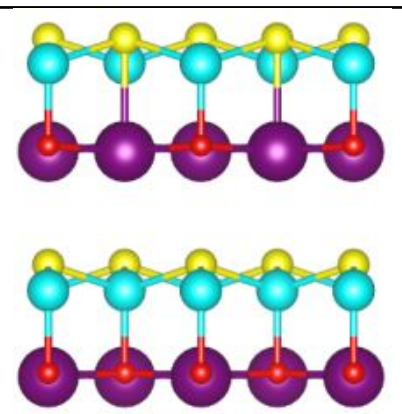	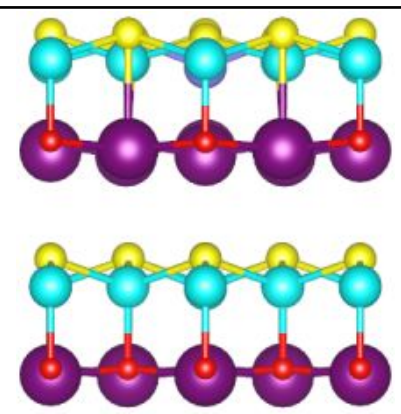	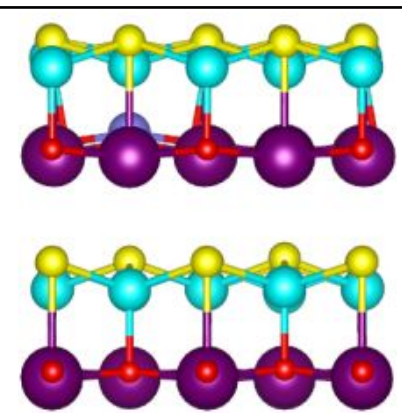	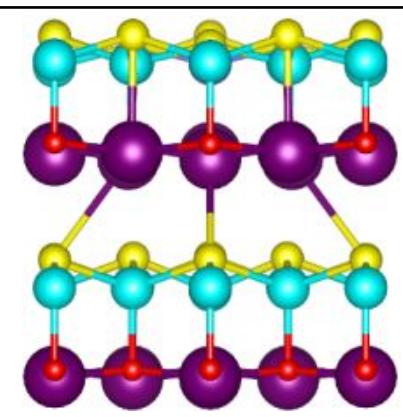	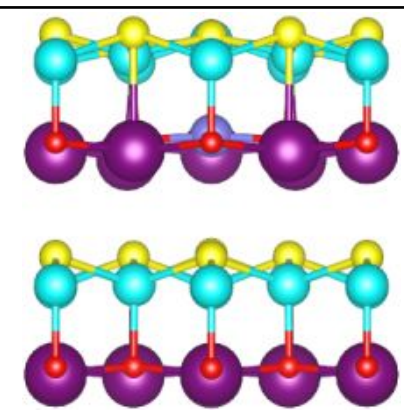	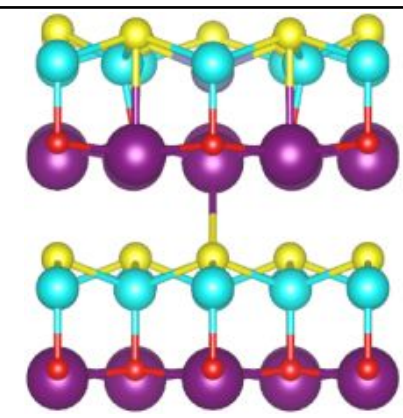	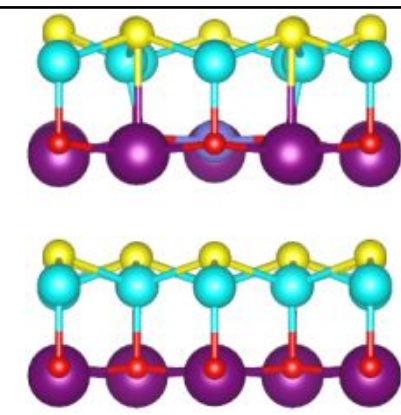

Table S4. The optimized adsorption structures of the NRA process over the relaxed Mn-CaZnOS catalyst.

$*NO_3$	$*NO_2$	$*NO$	$*N$	$*NH$	$*NH_2$	$*NH_3$
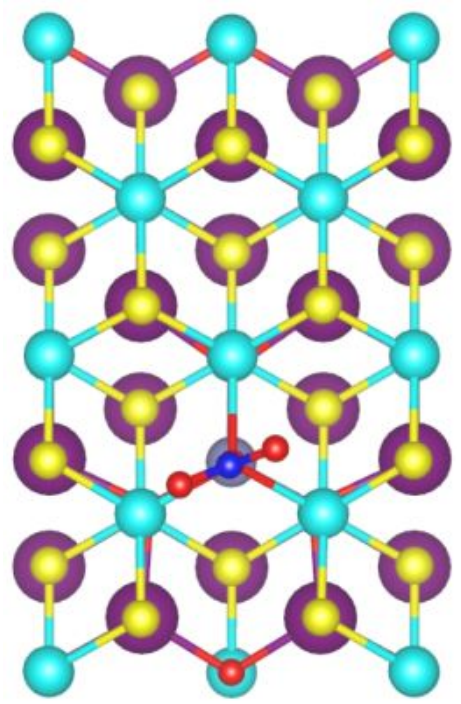	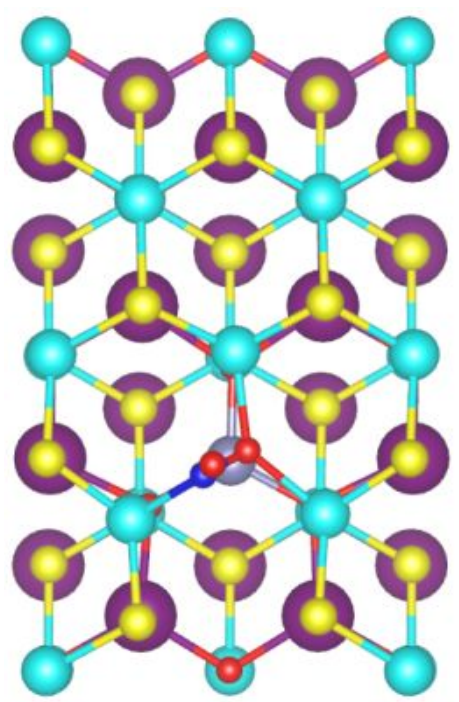	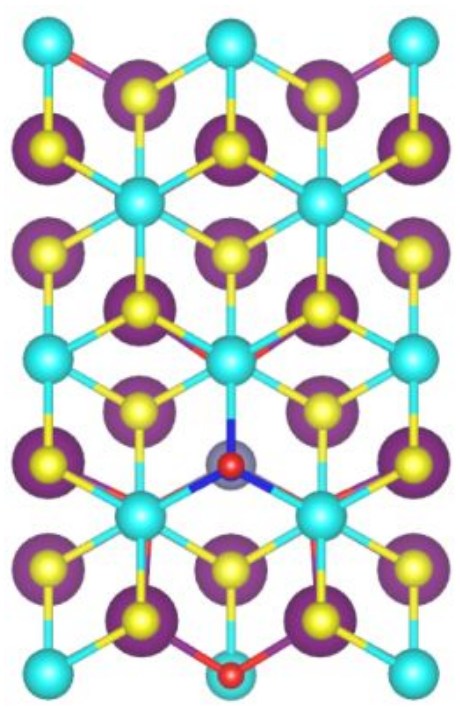	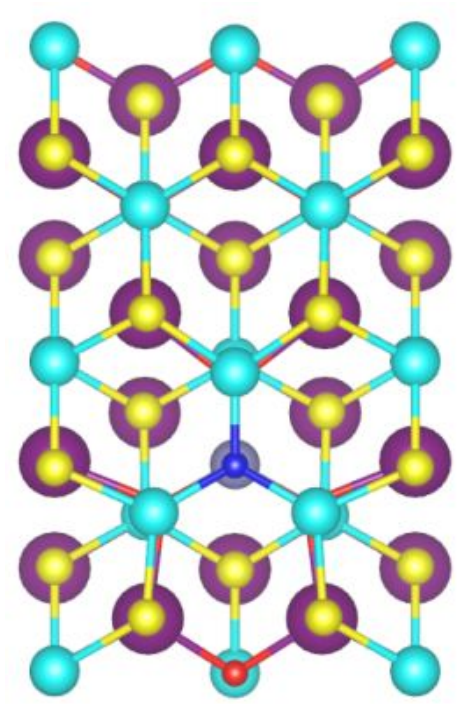	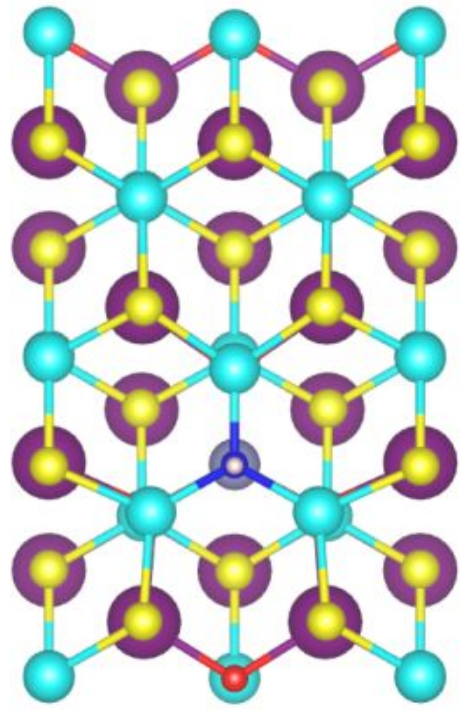	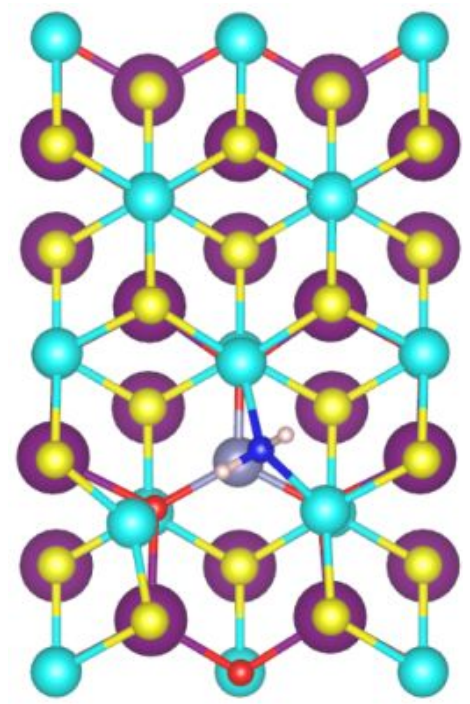	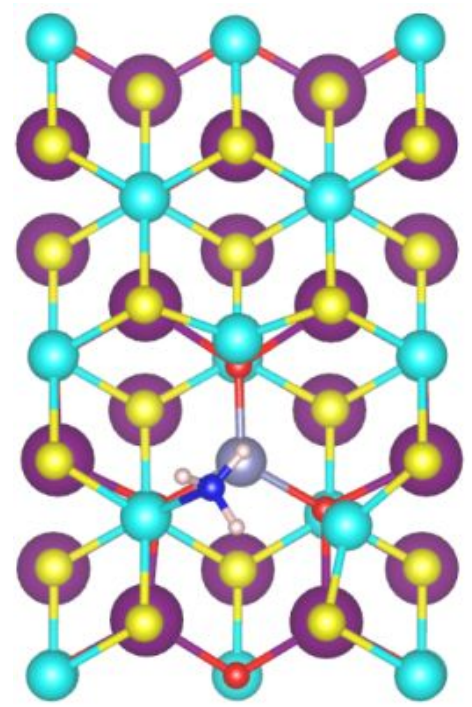

Table S5. The optimized adsorption structures of the NRA process over the pressed Mn-CaZnOS catalyst.

$*NO_3$	$*NO_2$	$*NO$	$*N$	$*NH$	$*NH_2$	$*NH_3$
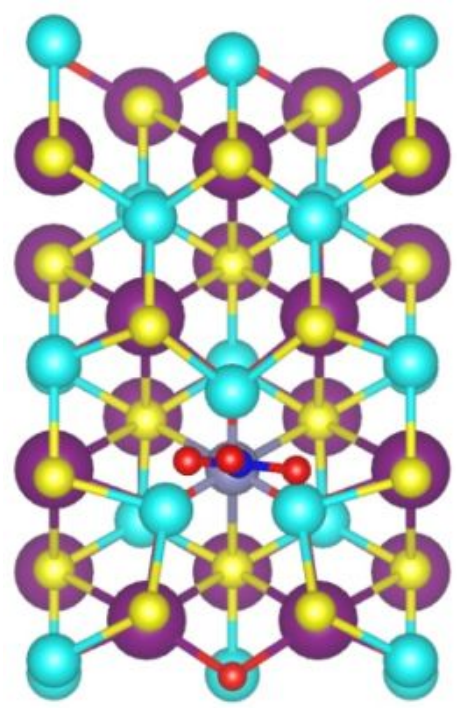	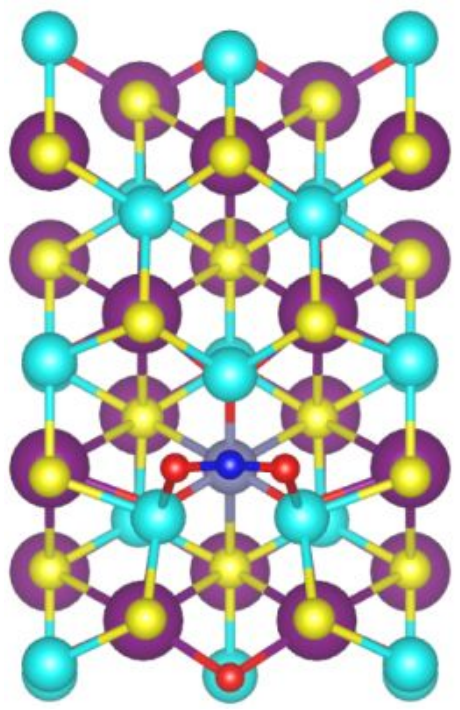	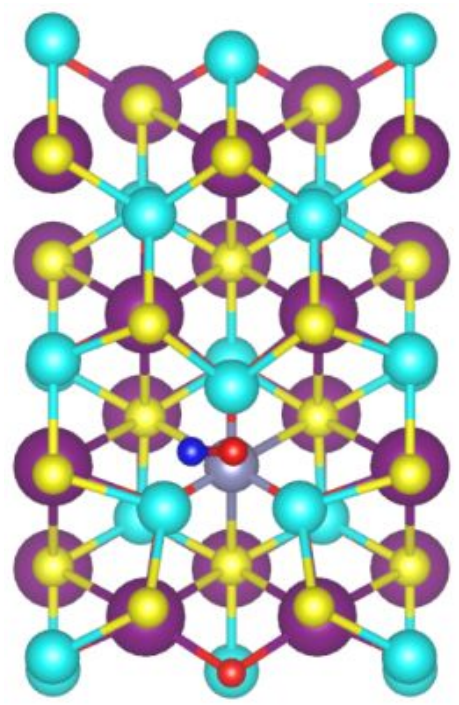	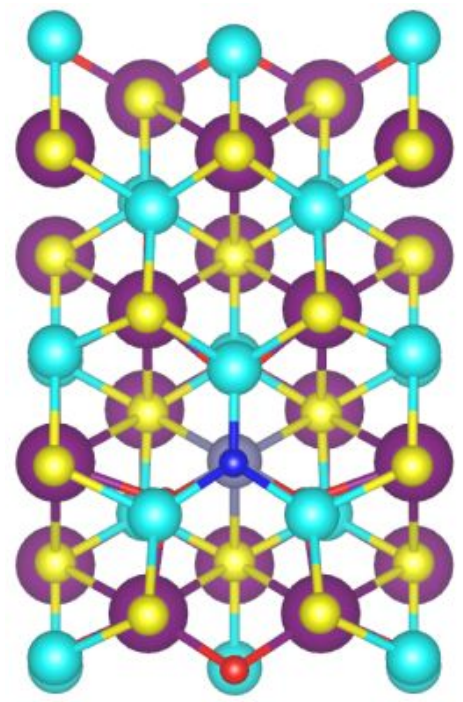	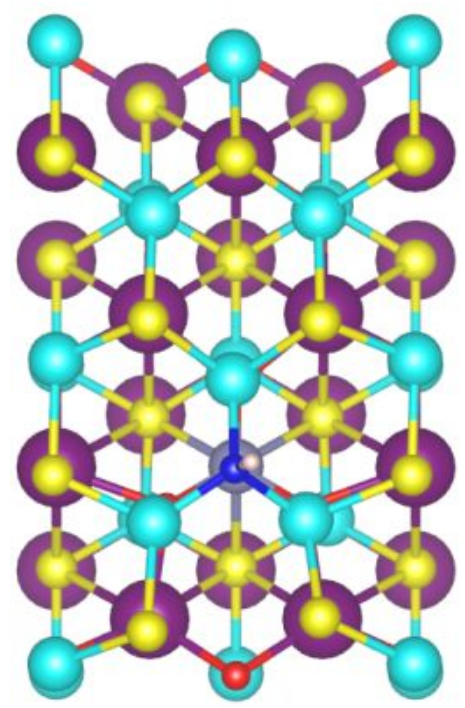	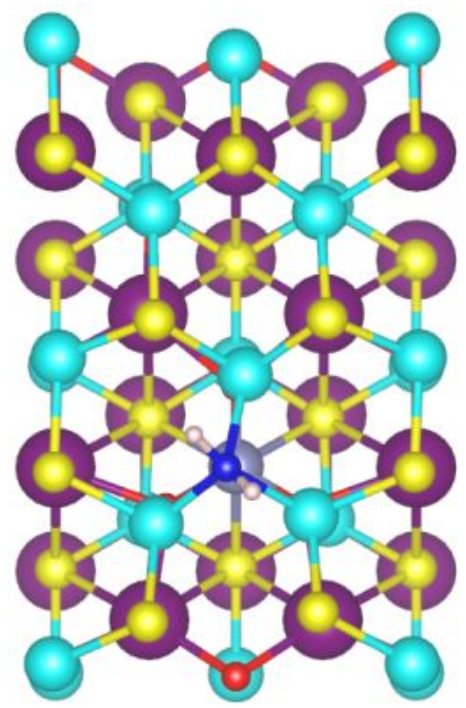	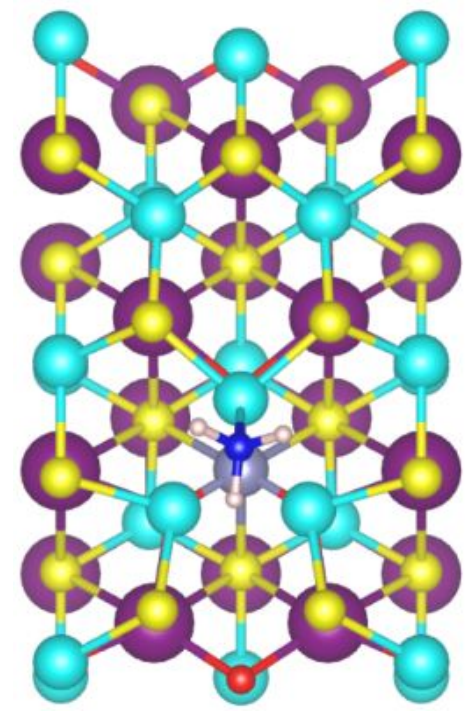
